# Community composition of phytopathogenic fungi significantly influences ectomycorrhizal fungal communities during subtropical forest succession

**DOI:** 10.1007/s00253-023-12992-5

**Published:** 2024-01-10

**Authors:** Meirong Chen, Jiazhi Yang, Chunquan Xue, Tieyao Tu, Zhiyao Su, Hanhua Feng, Miaomiao Shi, Gui Zeng, Dianxiang Zhang, Xin Qian

**Affiliations:** 1 Key Laboratory of Plant Resources Conservation and Sustainable Utilization, South China Botanical Garden, Chinese Academy of Sciences, Guangzhou, China; 2 University of Chinese Academy of Sciences, Beijing, China; 3 Guangdong Forestry Survey and Planning Institute, Guangzhou, China; 4 South China Agriculture University, Guangzhou, China; 5 College of Life Sciences, China West Normal University, Nanchong, China; 6 College of Life Sciences, Fujian Agriculture and Forestry University, Fuzhou, Fujian China

**Keywords:** Ectomycorrhizal fungi, Phytopathogenic fungi, Forest successions, Fungal community

## Abstract

**Abstract:**

Ectomycorrhizal fungi (EMF) can form symbiotic relationships with plants, aiding in plant growth by providing access to nutrients and defense against phytopathogenic fungi. In this context, factors such as plant assemblages and soil properties can impact the interaction between EMF and phytopathogenic fungi in forest soil. However, there is little understanding of how these fungal interactions evolve as forests move through succession stages. In this study, we used high-throughput sequencing to investigate fungal communities in young, intermediate, and old subtropical forests. At the genus level, EMF communities were dominated by *Sebacina*, *Russula*, and *Lactarius*, while *Mycena* was the most abundant genus in pathogenic fungal communities. The relative abundances of EMF and phytopathogenic fungi in different stages showed no significant difference with the regulation of different factors. We discovered that interactions between phytopathogenic fungi and EMF maintained a dynamic balance under the influence of the differences in soil quality attributed to each forest successional stage. The community composition of phytopathogenic fungi is one of the strong drivers in shaping EMF communities over successions. In addition, the EMF diversity was significantly related to plant diversity, and these relationships varied among successional stages. Despite the regulation of various factors, the positive relationship between the diversity of phytopathogenic fungi and EMF remained unchanged. However, there is no significant difference in the ratio of the abundance of EMF and phytopathogenic fungi over the course of successions. These results will advance our understanding of the biodiversity–ecosystem functioning during forest succession.

**Key points:**

•*Community composition of both EMF and phytopathogenic fungi changed significantly over forest succession.*

•*Phytopathogenic fungi is a key driver in shaping EMF community.*

•*The effect of plant Shannon’s diversity on EMF communities changed during the forest aging process.*

**Supplementary Information:**

The online version contains supplementary material available at 10.1007/s00253-023-12992-5.

## Introduction

Soil biodiversity plays a pivotal role in maintaining ecosystem services including nutrient cycling and plant production (Delgado-Baquerizo et al. 2020). A positive correlation has been observed between soil microbial diversity and multifunctionality in terrestrial ecosystems (Delgado-Baquerizo et al. 2016). Ectomycorrhizal fungi (EMF) are a key component of the forest soil microbiome and a driving force in forest development (Anthony et al. 2022). Most ectomycorrhizal fungi (EMF) form mycorrhizae with plant hosts (Bruns et al. 2002), enabling the hosts to access nutrients and water from the soil (Read and Perez-Moreno 2003). At the same time, these fungi depend on plants for their organic carbon supply, meaning that plants drive fungal community assembly (Peay et al. 2010a, b; Sugiyama et al. 2008). Diverse plant assemblages can attract diverse EMF assemblages because these fungi are attracted to particular hosts (Ishida et al. 2007). As various plant species disperse, they provide microbes with novel harbors to forge symbionts (Mitchell et al. 2006; Richardson et al. 2000). In addition, EMF are also involved in the interactions between plants and other soil microorganisms, such as pathogens and mycorrhizosphere mutualists that produce nutrient element and guard against antagonists (Tedersoo et al. 2020). Therefore, EMF can drive plant community ecology, especially in forest ecosystems, by influencing belowground traits of plants, altering soil processes and regulating plant coexistence (Tedersoo et al. 2020). Previous studies have observed that the EMF community varies over the course of forest succession (Yang et al. 2020; Gao et al. 2015; Twieg et al. 2007). Changes in the EMF community could lead to alterations in the multifunctionality of forest ecosystems. Despite this well-known phenomenon, the mechanisms underlying EMF community succession and their specific functions are poorly understood. Hence, examining species coexistence among fungal functional groups can help us gain insight into the processes that drive ecosystem functioning.

Plant host diversity tends to increase the diversity of EMF (Kernaghan et al. 2003). The EMF and host plant species have a very specific relationship, so any alteration in the plant species diversity will have a considerable impact on the functional traits of the symbiotic EMF community related to foraging (Reis et al. 2018). However, other facets of their relationship can vary across successional stages (Ishida et al. 2007). Both host (Yang et al. 2023; Roy et al. 2013; Tedersoo et al. 2008) and non-host plants (Taniguchi et al. 2007) can influence the EMF community structure, including their mechanisms to prevent pathogenic infections (Hortal et al. 2017; Sachs et al. 2010). Host preference and its restricted distribution significantly affect the EMF colonization, and due to the limited availability of habitats, the establishment of EMF communities is contingent on encountering compatible partners early (Myers and Harms 2009, Põlme et al. 2018). Therefore, the EMF community structure can also be significantly impacted by other soil microorganisms that coexist with them. Pathogenic fungi are also thought to be influential in maintaining the diversity of plant communities via host-specialized effects (Wang et al. 2019), and may have an effect on EMF diversity, as EMF are known to modify the susceptibility of hosts to phytopathogenic fungi (Buée et al. 2009; Lambers et al. 2018). The EMF form a mantle and Hartig net around the roots providing effective physical obstacles to the infiltration or propagation of pathogens into the roots, and can release antibiotic compounds in the mycorrhizosphere to inhibit pathogen sporulation (Laliberté et al. 2015). Laliberté et al. (2015) demonstrated that the antagonistic relationship between EMF and pathogens may explain the occurrence of monodominant stands of ECM plants among otherwise species-rich communities. EMF can reduce accumulation rates of pathogens in tree roots (van der Putten 2017), offsetting the negative plant–soil feedback (PSFs) induced by pathogen (Chen et al. 2019; Liang et al. 2020). Yang et al (2021) demonstrated that EMF shifts were heavily impacted by the variety of other fungal guilds, which will, in turn, provide feedback to plant community succession through fungal-associated biogeochemical processes.

Effective partners receive more positive feedback from their symbionts (Hortal et al. 2017). Plants in a relationship with mycorrhizal fungi use various strategies to access nutrients (Brundrett and Tedersoo 2018; Lambers and Teste 2013). EMF can reduce competition among plants, equalizing the distribution of nutrition among different plant species, which can help foster their coexistence (Perry et al. 1992). Soil factors have a major impact on this distribution regulation. Soil carbon/nitrogen (C/N) ratio (Han et al. 2017; Miyamoto et al. 2014), soil calcium (Põlme et al. 2013), N availability (Cox et al. 2010), and elevation (Bahram et al. 2012) can also greatly impact EMF diversity. Recently, Labouyrie et al. (2023) found that ectomycorrhizal fungi were usually correlated with soils that had a low pH, a low phosphorus content, and a high percentage of coarse fragments. In addition, soil properties (e.g., soil conductivity) can also influence the relationships between ECM and other fungal functional groups (Yang et al. 2021). Although recent improvements in sequencing and computational tools have allowed us to gain more insight into these complex interactions, we are still uncertain of the degree to which biotic and abiotic factors structure EM fungal communities during forest succession. In this study, we analyze soils collected from different stages of forest succession to (1) illuminate relationships between EMF and phytopathogenic fungal communities and (2) determine the effect of soil properties and plants on the EMF communities. Investigating how EMF and phytopathogenic fungi interact at various stages of forest succession could provide insights into the factors driving microbial interactions. We hypothesize that plant host diversity and soil properties mediate the interaction between the EMF community and phytopathogenic fungi during forest succession.

## Materials and methods

### Study sites and predictor factors

We surveyed and sampled 400 m^2^ (20 m × 20 m) subplots in 48 subtropical secondary forests located in southern Chinese nature reserve. Specifically, these sites were in four counties in the province Guangdong (Table S1): RuYuan (RY, 9 sites), YingDe (YD, 12 sites), YangShan (YS, 12 sites), and LeChang (LC, 15 sites). The vegetation in these sites was comprised of typical subtropical evergreen broad-leaf forests at different successional stages, and we identified 24 species (Table S2) from eight families as hosts for EMF according to Tedersoo and Smith (2013). These species were *Quercus fabri*, *Castanea* sp., *Castanea mollissima*, *Platycladus orientalis*, *Castanopsis fissa*, *Castanopsis hystrix*, *Cyclobalanopsis glauca*, *Quercus aliena*, *Fagus longipetiolata*, *Castanopsis fargesii*, *Castanopsis sclerophylla*, *Castanopsis lamontii*, *Castanopsis faberi*, *Cyclobalanopsis stewardiana* (*Fagaceae*), *Tilia tuan* (*Malvaceae*), *Carpinus turczaninowii* (*Betulaceae*), *Taxus wallichiana* var. *chinensis*, *Cephalotaxus* *fortune* (*Taxaceae*), *Pinus massoniana*, *Pinus elliottii* (*Pinaceae*), *Cunninghamia lanceolata* (*Cupressaceae*), *Trachycarpus* *fortune* (*Arecaceae*), and *Rhodomyrtus tomentosa* (*Myrtaceae*)*.* Sampling sites were categorized into three groups according to forest successional stages (young, 0–24 years; intermediate, 25–35 years; and old, > 35 years) (Table S1). Totally, there were 12, 15, and 21 plots in these respective groups.

Soil samples were collected from the topsoil layer (15-cm depth). After sieving the samples, we placed fine soils in plastic bags and stored them at − 80 °C until DNA extraction. In total, 240 soil samples were collected. In addition, we measured the depth of litterfall and the following 17 soil properties: humic substance, organic matter, total N, total P, pH, total K, available N, available P, exchangeable K, exchangeable Ca, exchangeable Mg, available Cu, available Zn, available Fe, available Mn, available B, and water content (Table S3). For each plot, we recorded slope, elevation, and plant species (Table S1 and S2).

### Amplification and sequencing

DNA was extracted from each of the soil samples using TGuide S96 Magnetic Soil/Stool DNA Kit (cat. DP812, TIANGEN, Beijing, China) following the manufacturer’s instructions. The ITS region of extracts was amplified in an ABI Applied Biosystems Veriti 96-well using the primers ITS2F: GCATCGATGAAGAACGCAGC and ITS2R: TCCTCCGCTTATTGATATGC. Fragments were denatured at 95 °C for 5 min, then 25 cycles at 95 °C for 1 min, 50 °C for 30 s, 72 °C for 40 s, and a final extension at 72 °C for 7 min. Each sample was amplified in triplicate to minimize PCR biases, and the replicate products were pooled together into a general sample. The final PCR products were purified using the e.Z.N.A.™ Cycle-Pure Kit (Omega, Beijing, China). Illumina TruSeq DNA PCR-Free Library Preparation Kit (Illumina, USA) was used to construct sequencing libraries as per the manufacturer’s protocol with index sequence. Subsequently, Qubit 2.0 Fluorometer (Thermo Scientific) was employed to evaluate the library quality. Sequencing of the qualified libraries was conducted on the Illumina novaseq6000 (Illumina, USA).

### Molecular analysis

USEARCH version 9 (Edgar 2010) was used to demultiplex and remove low-quality sequences. Operational taxonomic unites (OTUs) were generated at 97% sequence similarity cutoff employing the UPARSE (Edgar 2013) pipeline in the same program. A representative sequence of each OTU was picked and searched against the UNITE reference database (version 7.2, Nilsson et al. 2019) using a BLAST algorithm to check identity of fungal OTUs. RDP Classifier was employed to assign taxonomic classification to each OTU, with a cutoff of 0.8 to eliminate uncertain assignments. To eliminate the effects of read number variation from the different plots on downstream analysis and analyze relationship between EMF community and pathogen fungal community at the same sequencing depth, the number of sequences of each sample was rarefied to an even depth (24,504) using the command shared in QIIME 2 (Bolyen et al. 2019). FunGuild (Nguyen et al. 2016) was applied to assign OTUs to identify EMF and phytopathogenic fungi.

### Statistical analysis

Most statistical analyses were performed on R 4.1.3. A Kruskal test followed by a Wilcoxon test was performed to detect alpha diversity among different successional stages. Principal coordinate analysis (PCoA) was used to show community differentiation of whole fungi, EMF, and phytopathogenic fungi at each successional stage, based on Bray–Curtis distance using “ape” R package (Paradis and Schliep 2018). The permutational multivariate analysis of variance (PERMANOVA) and Adonis analysis with 999 permutations were used to compare the fungal community beta diversity among three groups. Additionally, we calculated Shannon’s diversity indices for herbs, associated trees, trees, tree width (*W*), associated tree *W*, ECM trees, and all plants. Finally, we reviewed 27 factors (including biotic and abiotic factors, Table S1 and S3) which were divided into three categories: (1) plant factors, (2) soil properties, and (3) environmental factors.

The *P* values of comparisons were corrected using Bonferroni correction. Kruskal tests were applied to richness and Shannon’s diversity indices to determine significant differences for plant alpha diversity. We performed these analyses for each successional stage (1) with all hosts included, (2) herbs only, and (3) companion species only. The “amplicon” R package was used to display the composition of the EMF and phytopathogenic fungal communities (https://github.com/microbiota/amplicon). The ratio of EMF relative abundance to pathogen was visualized using the same method as EMF and pathogen composition. Linear discriminant analysis effect size (LEfSe, http://huttenhower.sph.harvard.edu/galaxy/) was used to find significantly different biomarkers for each of these forest groups using the linear discriminant analysis (LDA) principle. We used 3.0 as the threshold to discriminate indicator features.

A Mantel test was performed to analyze and visualize the relationship among all factors and the EMF or pathogen fungal community in different groups using “tidyverse” (https://www.rdocumentation.org/packages/tidyverse/versions/1.3.2) and “ggcor” (https://rdrr.io/github/houyunhuang/ggcor/f/) R packages. We parsed out the effects of important factors found in the Mantel test, and then explored the independent influence of factors on EMF and pathogen community by employing partial Mantel tests. Multiple regression on dissimilarity matrices (MRM) was performed to explore the impact of soil, plant, and fungi (EMF or pathogen) on the composition of EMF or phytopathogenic fungi from different successional stages of forests using the “ecodist” R package (Goslee and Urban 2007). The MRM models were simplified using the result of “Hmisc” R package (https://github.com/harrelfe/Hmisc) which shows dependence among factors—until P < 0.05 for all distance matrices. Multiple linear regression was conducted to explore the effect of all predictive factors on EMF or pathogen relative abundance using “stats” R package. The optimal models were selected using “MuMIn” R package (https://rdrr.io/cran/Hmisc/), and standardized coefficients were obtained using “effectsize” R package (Ben Shachar et al. 2020).

## Results

### Data characteristics

Each forest was dominated by different plant species (Fig. S1). There was no statistical difference in tree diversity across successional stages (Fig. S2-7). The Shannon’s diversity index for herbs was not significantly different across successional stages (Fig. S8 and S9), but a significant difference was detected in herb richness between young and intermediate forests (Fig. S8).

**Fig. 1:**
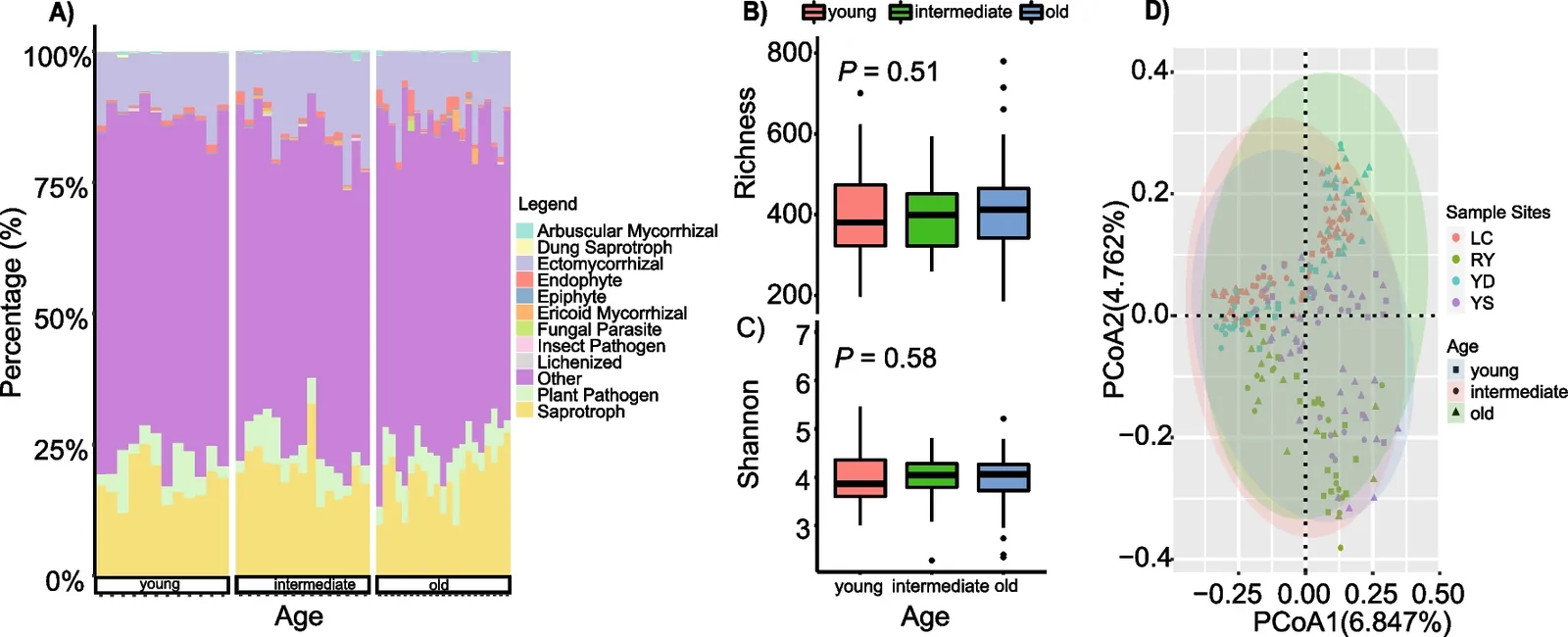
**A** Function-level distribution of fungi in 48 subtropical secondary forests. Lilac and green represent ectomycorrhizal fungi (EMF) and phytopathogenic fungi, respectively. **B, C** Box plots of the richness and Shannon’s diversity index, respectively, of all fungi species among young, intermediate, and old forest groups using Kruskal–Wallis test, and indicate no significant difference between forest groups. **D** Principal coordinate analysis (PCoA) plot for all fungi, which illustrates how fungal communities are structured along different successions based on Bray–Curtis distance. Shaded ellipses represent standard error of the mean (95%) and are colored by forest age (blue = young, pink = intermediate, green = old)

A saturation plateau in the rarefaction curves of the fungal OTUs revealed that vast majority of fungal diversity had been observed (Fig. S10). There are 2310 OTUs: 177 OTUs were identified as EMF, 168 OTUs were phytopathogenic fungi, and the rest were saprophytic fungi, arbuscular mycorrhizal fungi, endophytic fungi, and others. The reads number per sample ranged from 24,504 to 75,951, so the feature table was normalized to 24,504 (reads number from 14,974,560 to 5,880,960 after rarefying the feature table). Across the 48 sites, the relative abundance of EMF ranged from 5.51 to 26.24%, and phytopathogenic fungi varied from 2.35 to 14.59% (Fig. 1A). EMF OTUs belonged to 12 orders, 24 families, and 31 genera, while pathogen OTUs belonged to 22 orders, 41 families, and 69 genera.

### Fungal diversity throughout forest succession

**Fig. 2:**
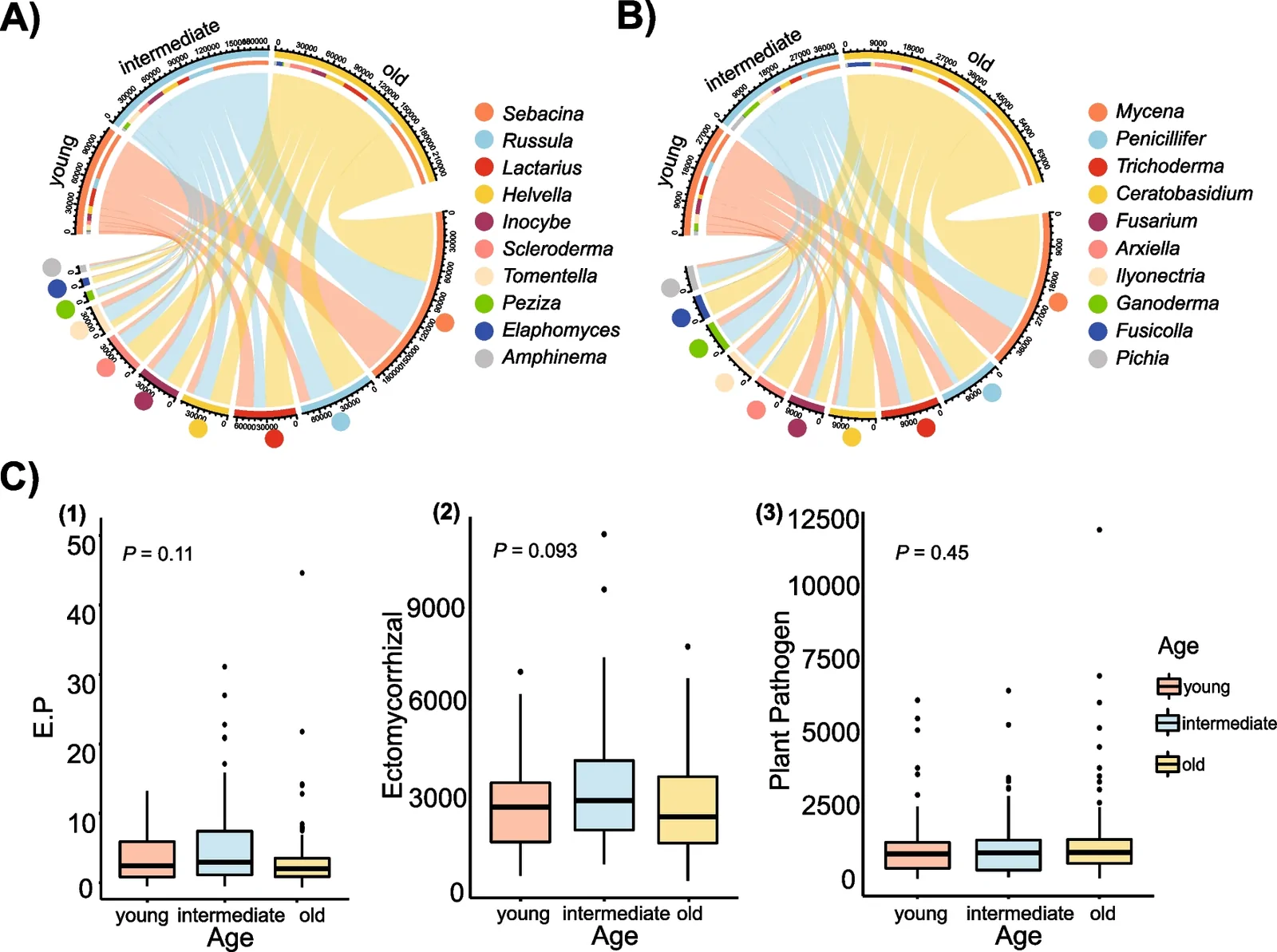
**A, B** The ten most abundant genera in ectomycorrhizal fungi (EMF) and phytopathogenic fungal composition, respectively. **C** (1) The variation of ratio of EMF and phytopathogenic fungal relative abundance (E.P) among young, intermediate, old forests (*P* = 0.11, > 0.05). (2) and (3) EMF and phytopathogenic fungal relative abundance, respectively, across different successional stages using Kruskal–Wallis test

**Fig. 3:**
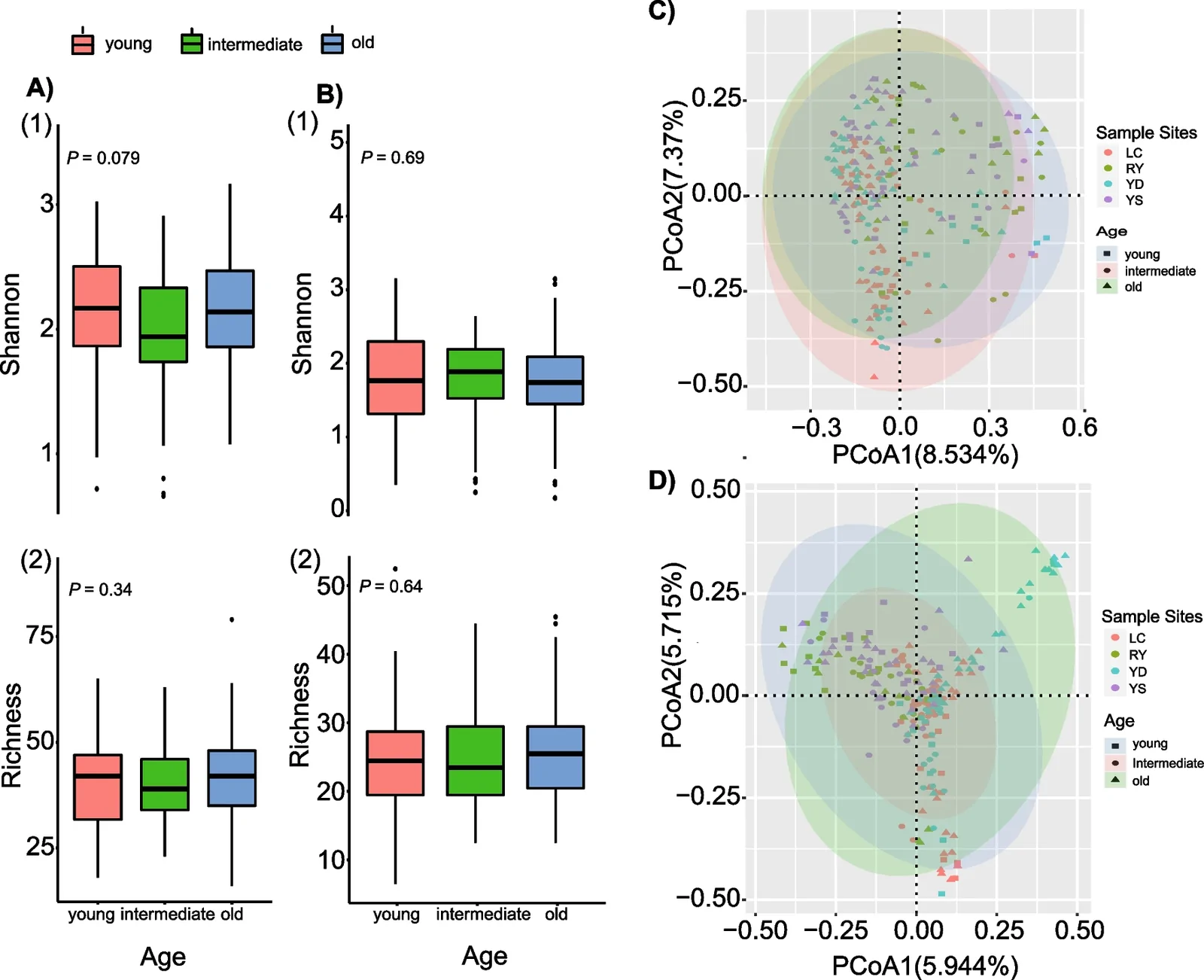
**A** Ectomycorrhizal fungi (EMF) Shannon’s diversity index (*P* = 0.079, > 0.05) and richness (*P* = 0.34, > 0.05) across forest successional stages using Kruskal–Wallis test. **B** Phytopathogenic fungal Shannon’s diversity index (*P* = 0.69, > 0.05) and richness (*P* = 0.54, > 0.05) difference along forest successions using Kruskal–Wallis test. **C, D** Unconstrained principal coordinate analysis (PCoA) with Bray–Curtis dissimilarity to display how EMF community and phytopathogenic fungal communities vary, respectively, over successional stages

For all fungi, there was no significant difference in alpha diversity (Fig. 1B, C) across successional stages. However, there were significant differences in their community compositions (Table 1, Fig. 1D). For the EMF communities (Fig. 2A), *Sebacina* was the dominant genus throughout the forests. Additionally, there was a high relative abundance of *Lactarius*, *Inocybe*, and *Russula* in young, intermediate, and old forests, respectively. Pathogen fungal communities were dominated by the genus *Mycena*, followed by *Penicillifer*, *Trichoderma*, and *Ceratobasidium* (Fig. 2B). There were no significant differences between EMF relative abundance, phytopathogenic fungi relative abundance, and the ratio between the two across different successional stages (Fig. 2C). To further analyze the EMF and phytopathogenic fungi communities from different successional stages, we compared the fungal alpha diversity and visualized their dissimilarity along different stages. While richness and Shannon’s diversity index for these stages did not vary significantly (Fig. 3A, B), EMF and phytopathogenic fungi communities displayed significant variation across successional stages (Fig. 3C, D). The differences between fungal communities were statistically confirmed (Table 1). LDA results showed that high relative abundance fungi were not the marker species. *Phaeosphaeriaceae*, *Sympodiella*, *Hypocreaceae*, *Thelonectria blackeriella*, *Leucoagaricus*, *Calvatia*, and *Pulveroboletus* are more abundant in young forests; *Minimelanolocus*, *Clavicipitaceae*, *Atractium*, *Amanita citrina*, *Malassezia globosa*, and *Mortierella nantahalensis* were significantly discriminant taxa in intermediate forests, whereas *Archaeorhizomycetales*, *Sporormiaceae*, *Cladorrhinum*, and *Chytridiales* responded significantly in old forests (Figure S11).

**Table 1:** Permutational multivariate analysis of variance (PERMANOVA) of the community composition of all fungi, as well as the ectomycorrhizal fungi (EMF) and phytopathogenic fungi separately for each forest successional stage

Parameter	Pairs	Sums of sqs	*F*	*R*^2^	*P*
All fungi	**Young/old**	1.2721	3.2229	0.0194	**
	**Young/intermediate**	0.9826	2.4864	0.0184	**
	**Old/intermediate**	1.2949	3.1935	0.0176	**
EMF	**Young/old**	1.1208	2.7466	0.0166	**
	**Young/intermediate**	1.1873	2.8995	0.0213	**
	**Old/intermediate**	1.5386	3.7136	0.0204	**
Phytopathogenic fungi	**Young/old**	1.0581	2.3684	0.0143	**
	**Young/intermediate**	0.9432	2.1195	0.0157	**
	**Old/intermediate**	1.3349	2.9718	0.0164	**

^**^0.001 < *P* < 0.01

### Factors influencing the relative abundance of EMF and phytopathogenic fungi throughout forest succession

EMF relative abundance was influenced by various factors depending on the stage of forest succession (Table 2). In the young forest group, EMF abundance was significantly influenced by pH (coefficient = 0.8, *P* = 3.53e − 3) and water content (coefficient =  − 0.78, *P* = 4.28e − 3). In the intermediate forest group, EMF abundance was significantly influenced by plant (plant Shannon’s diversity: coefficient =  − 0.72, *P* = 4e − 04; ECM tree Shannon’s diversity: coefficient = 0.51, *P* = 7.6 e − 03) and soil factors (exchangeable Mg: coefficient =  − 0.77, *P* = 0.003; pH: coefficient = 0.77, *P* = 0.011; quick acting K: coefficient = 1.45, *P* = 0.000), especially plant diversity. Soil properties had varying effects on EMF abundance. In the old forest group, however, EMF abundance was only influenced by soil and environmental factors, especially available N (coefficient = 1.18, *P* = 1.20e − 08) and pH (coefficient = 0.78, *P* = 3.05e − 05).

**Table 2:** Multiple linear regression shows the relationship between ectomycorrhizal fungi (EMF) abundance and abiotic (soil and environmental) and biotic (plant) factors in different forest successional stages

Respondent	Forest age	Parameter	Coefficient	*t*	*P*	Sig	Model parameters
EMF abundance	Young	pH	0.8	3.044	0.0035	**	*R*^2^_adj_ = 0.1145, *F* (2, 57) = 4.816, *P* = 0.0117
		Water content	− 0.78	− 2.976	0.0043	**	
	Intermediate	Plant Shannon’s diversity	− 0.72	− 3.74	0.0004	***	*R*^2^_adj_ = 0.3802, *F* (6, 68) = 8.566, *P* = 6.128e − 07
		ECM tree Shannon’s diversity	0.51	2.751	0.0076	**	
		Exchangeable Mg	− 0.77	− 3.079	0.0030	**	
		pH	0.77	2.606	0.0112	*	
		Quick acting K	1.45	5.266	0.0000	***	
		Total K	− 0.96	− 3.452	0.0010	***	
	Old	Available N	1.18	6.214	0.0000	***	R^2^_adj_ = 0.272, *F* (4,100) = 10.71, *P* = 2.886e − 07
		elevation	− 0.29	− 2.503	0.0139	*	
		Litterfall	0.27	2.367	0.0199	*	
		pH	0.78	4.369	0.0000	***	
E/P	Young	Humic substances	− 0.95	− 3.773	0.0004	***	R^2^_adj_ = 0.2531, *F* (4,55) = 5.999, *P* = 0.0004
		Plant Shannon’s diversity	0.62	3.32	0.0016	**	
		Total K	− 1.11	− 3.916	0.0003	***	
		Total N	− 1.11	3.151	0.0026	**	
	Intermediate	Plant Shannon’s diversity	− 0.5	− 3.727	0.0004	***	*R*^2^_adj_ = 0.3195, *F* (4,70) = 9.684, *P* = 2.719e − 06
		ECM tree Shannon’s diversity	0.31	2.269	0.0264	*	
		Litterfall	0.4	3.117	0.0027	**	
		Total N	0.6	4.618	0.0000	***	
	Old	slope	0.21	2.188	0.0310		*R*^2^_adj_ = 0.0628, *F* (2, 102) = 4.485, *P* = 0.0136
						*	
		Total N	0.17	1.828	0.0704		

^***^P ≤ 0.001; **0.001 < *P* ≤ 0.01; *0.01 < *P* ≤ 0.05

Contrary to our hypothesis, there were no significant relationships between EMF relative abundance and pathogen fungal relative abundance between each of the successional stages. While the results were not significant, there was a trend in young forests indicating that the ratio of EMF to pathogen relative abundance (E/P) might be influenced by plant factors and soil properties (Table 2). The E/P in younger forests was influenced to a greater extent and by more complex factors than the older group. However, the only factors to significantly influence E/P in old stage were slope (coefficient = 0.21, *P* = 0.031) and total N (coefficient = 0.17, *P* = 0.07) in old forests.

We also explored the effects of various factors on the relative abundance of the phytopathogenic fungi in different forest stages (Table S4). During the course of forest succession, the relative abundance of the phytopathogenic fungi was influenced by various factors, with soil properties consistently exerting a significant influence. Notably, in old forests, the phytopathogenic fungi appeared to be largely independent of plant influence, being primarily affected by soil factors and slope (available *B* = 0.17, exchangeable Ca = 0.22, slope =  − 0.21, all *P* < 0.05). Conversely, in young forests, plants exhibited a direct and statistically significant effect on the relative abundance of the phytopathogenic fungi (ECM tree Shannon’s diversity =  − 0.65, plant Shannon’s diversity =  − 0.52, all *P* < 0.05). In intermediate forests, both litterfall and total N were found to be inversely related to phytopathogenic fungi relative abundance (litterfall =  − 0.35, total N =  − 0.46, all *P* < 0.05). The factors influencing the relative abundance of phytopathogenic fungi were different from those influencing the relative abundance of EMF over successions.

### Factors that affect EMF diversity in different forest successional stages

**Fig. 4:**
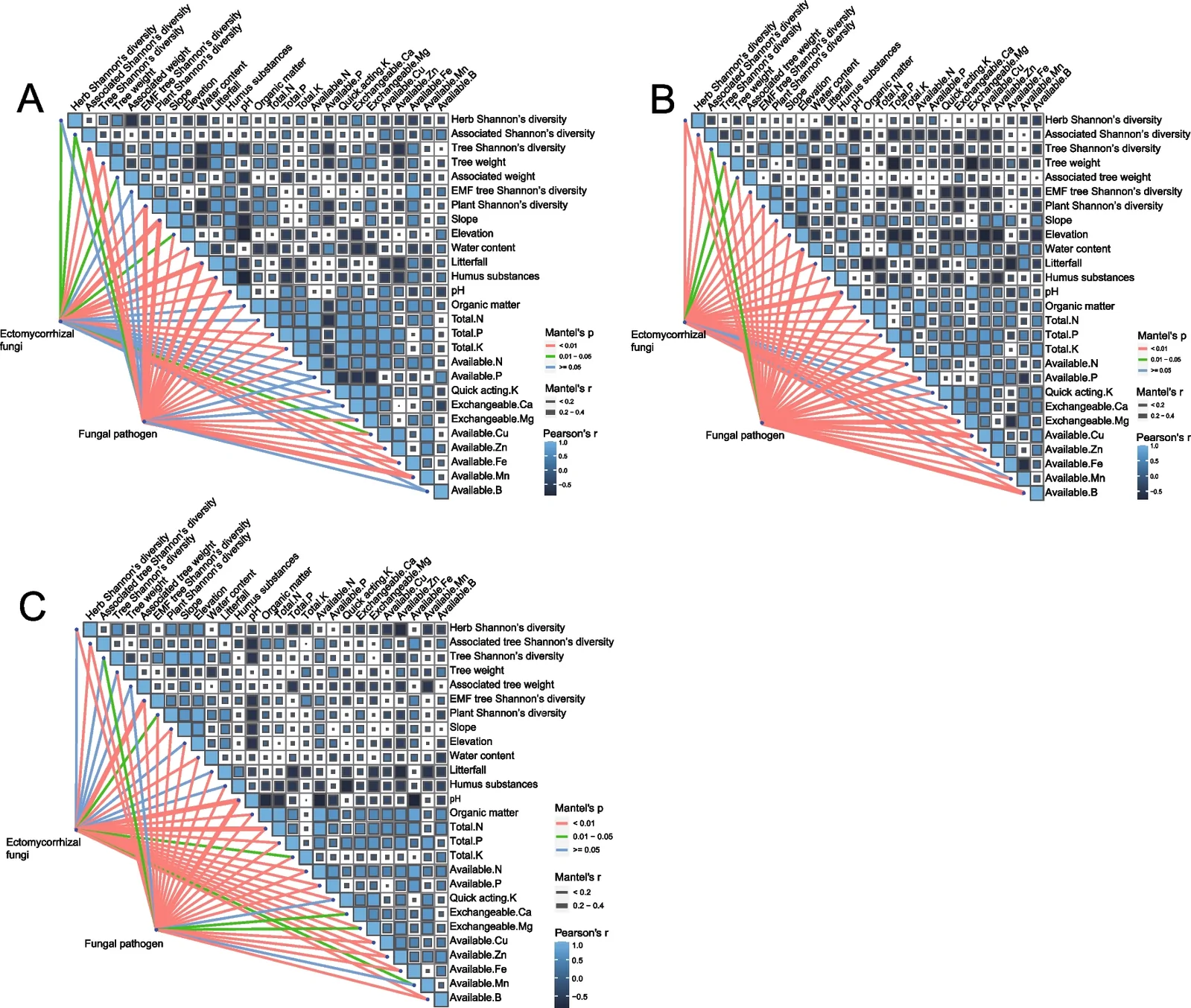
Biotic and abiotic drivers of EMF and phytopathogenic fungal community composition in young (**A**), intermediate (**B**), and old (**C**) forests. Pairwise comparisons between each factor are shown with a color gradient indicating Spearman’s correlation coefficients. EMF and phytopathogenic fungal community composition were related to each environmental factor using partial Mantel tests. Edges are colored by the statistical significance with 9999 permutations, edge width represents strength of the corresponding distance association

The EMF communities were impacted by different factors depending on the forest’s successional stage (Fig. 4). Plants, soil qualities, and environmental factors all had a significant impact on the community composition of EMF and phytopathogenic fungi in soil, while there was no significant association between EMF tree Shannon’s diversity in the young stage (Fig. 4). EMF communities were significantly related to various factors along different successions (all *P* < 0.05; Table S5). Comparing all factors, the MRM models (all *P* < 0.05, Table 3) indicate that pathogen diversity had a significant influence on EMF diversity regardless of the successional stage of the forest (young: coefficient = 0.0392, *P* = 0.0001; intermediate: coefficient = 0.328, *P* = 0.0001; old: coefficient = 0.0326, *P* = 0.0001). However, for young and intermediate forests in particular, plant factors (young: ECM tree Shannon’s diversity: coefficient =  − 0.0253, *P* = 0.0271, intermediate: tree Shannon’s diversity: coefficient = 0.0122, *P* = 0.0008), and herb diversity (coefficient = 0.0126, *P* = 0.0002), respectively, played a key role in increasing the diversity of EMF. For all the successional stages, the relative abundance of phytopathogenic fungi did not have a significant correlation with EMF relative abundance, but the E/P ratio was not significantly different between successional stages. EMF community composition was closely related to plant pathogen community across the stages of succession (Table S6: young, coefficient = 0.0345, *P* = 0.0001; intermediate, coefficient = 0.0256, *P* = 0.0001; old, coefficient = 0.0300, *P* = 0.0001). EMF diversity and phytopathogenic fungi diversity were the greatest predictors for each other across the forest successional stages.

**Table 3:** Multiple regression of distance matrices (MRM) of ectomycorrhizal fungi (EMF) community composition in relation to abiotic and biotic predictors for young, intermediate, and old forests

Responders	Forest age	Distance matrix	Coefficient	*P*	Sig	Model parameters
EMF	Young	ECM tree Shannon’s diversity	− 0.0253	0.0271	*	*R*^2^ = 0.2220, *F* = 100.6492, *P* = 0.0001
		Humic substances	0.0132	0.0047	**	
		Litterfall	0.0155	0.0015	**	
		Available Fe	0.0273	0.0168	*	
		Phytopathogenic fungi	0.0392	0.0001	***	
	Intermediate	Humic substances	0.0086	0.0020	**	*R*^2^ = 0.2165, *F* = 109.2268, *P* = 0.0001
		Elevation	− 0.0114	0.0008	***	
		pH	0.0150	0.0001	***	
		Available B	0.0088	0.0006	***	
		Herbs Shannon’s diversity	0.0126	0.0002	***	
		Tree Shannon’s diversity	0.0122	0.0008	***	
		Phytopathogenic fungi	0.0328	0.0001	***	
	Old	ECM tree Shannon’s diversity	0.0164	0.0001	***	*R*^2^ = 0.2286, *F* = 230.7766, *P* = 0.0001
		Slope	0.0064	0.0263	*	
		Elevation	− 0.0064	0.0203	*	
		pH	0.0092	0.0001	***	
		Available B	0.0084	0.0013	**	
		Available Cu	0.0060	0.0090	**	
		Phytopathogenic fungi	0.0326	0.0001	***	

^***^P ≤ 0.001; **0.001 < *P* ≤ 0.01; *0.01 < *P* ≤ 0.05

## Discussion

### Effect of plant and soil properties on abundance of EMF in successional stages

Plants are an important driver of the establishment of EMF communities and respond quickly to mycorrhizal colonization through various mechanisms (Gorzelak et al. 2015). Plant dispersal limitation restricted the assembly of EMF communities (Glassman et al. 2017; Peay et al. 2010a, b). In different stages, plants showed both direct and indirect effects on the relative abundance of EMF (Table 2). In young forests, plant diversity did not have an influence on the abundance of EMF. However, ECM tree diversity showed a positive effect on the relative abundance of EMF during the intermediate stage. Host trees serve as important islands for the EMF community’s colonization (Peay et al. 2007). There was a positive relationship between the diversity of mycorrhizal fungi and plant diversity, which may partly be caused by partner specificity in EMF (Hiiesalu et al. 2014). In old forests, EMF relative abundance was found to rise in correlation with increase in litterfall, suggesting that plants have an indirect effect on the abundance of EMF. Additionally, the pH of the soil has a major impact on the relative abundance of EMF during different successional stages. This is in line with the results of Põlme et al. (2013) regarding the biogeography of EMF associated with alders. The role of soil properties in forming EMF communities is undeniable; the regulation of soil properties is necessary to maintain the ecosystem services.

### E/P ratio across successional stages

No significant difference was observed in the ratio of relative abundance of EMF and phytopathogenic fungi (E/P) among the different stages, indicating a balance between EMF and phytopathogenic fungi across forest succession. Over successive periods, various factors have been identified as responsible for the stabilization of the forest ecosystem. Plant diversity played a role in regulating the E/P ratio in young and intermediate forests, whereas this was not the case in old stage forests (Table 2). One explanation is that the stability of the plant community is greater in old forests, which may offset the effects of plant diversity on soil fungi. In the early and middle stages, EMF and phytopathogenic fungi vied for the opportunity to colonize the partner, but this rivalry was not evident in an established, long-term ecosystem (McCormick et al. 2018).

Although the relative abundance of phytopathogenic fungi and EMF communities did not have a positive relationship with each other, they did reach a dynamic balance within each forest’s successional stage (Fig. 2C). Further, we found that there was a strong, positive interaction between EMF and the phytopathogenic fungi diversity that was maintained over the different forest stages. They kept the strongest linkage at the same level, with increasing pathogen fungal diversity explaining 3.920%, 3.278%, and 3.265% of the increase in EMF diversity in soils from young, intermediate, and old forests, respectively. This is in accordance with McGuire et al. (2006), who found that fungal taxa are functionally diverse in forest soils and that different fungi respond differently to soil substrate. Thus, the soil substrate may have consistently attracted particular species of phytopathogenic fungi and EMF, resulting in a positive relationship in their species composition. To this point, our results indicate that the diversity of phytopathogenic fungi increased as the diversity of EMF species increased. This positive relationship reflects a co-occurrence of biodiversity in EMF and phytopathogenic fungi communities, suggesting a functional difference during EMF community successions. EMF communities tend to show functional differences at the species level (Anthony et al. 2022). Our findings demonstrate that the pathogen community is a critical factor influencing EMF diversity under the regulation of plant diversity and soil properties, despite spatial heterogeneity. The rivalry between EMF and phytopathogenic fungi had a major influence on the fluctuating equilibrium between contenders, and this interaction was enhanced over successive periods through equalizing mechanisms (Tedersoo et al. 2020).

### Effect of plant diversity on EMF diversity throughout successional stages

Plants use several chemical mechanisms to resist pathogen infection (Oostendorp et al. 2001). When some plants are able to suppress infection and other are not, forests can move towards monodominant stands. In our study, plant variables were strong determinants of EMF community composition across successional stages, demonstrating that host selection remains an essential driver in shaping EMF community structure regardless of local abiotic conditions. Specifically, host diversity decreased EMF diversity in young forests, which did not occur in old or intermediate forests. This was corroborated by the host Shannon’s diversity index, which indicated a negative association between hosts and the EMF community in young forests. Similarly, increasing the EMF diversity contributed to the low diversity of host plants in young forests. Further, positive PSFs were more prevalent in young forests than in old and intermediate forests (Table S4). The soil characteristics found in young forests could in part explain these differences in microbial function (Ryan and Angus 2003; Xinpeng et al. 2020). Competition-colonization tradeoffs increase diversity in EMF communities (Schimel and Schaeffer 2012; Smith et al. 2018). This is because these tradeoffs encourage resource partitioning, causing various fungal groups to target different available substrates (Schimel et al. 2005). Increased N concentration in soil reduces the diversity of fungi (Allison et al. 2008, 2007). Finally, we found that the EMF community was affected differently by the various elements of each forest successional stage. Plant diversity had a greater influence on fungal communities than environmental factors did, regardless of the successional stage.

In conclusion, we studied the relationship between phytopathogenic fungal communities and EMF communities in soils collected from forests across different successional stages. Shifts in the EMF community in response to changing environment conditions imply that soil and biotic factors (including plant and microbial factors) have varying degrees of influence on EMF composition in different successional stages. However, the co-occurrence of these factors maintained the EMF community in a dynamic equilibrium of diversity and relative abundance. The same level of interaction between phytopathogenic fungi and EMF communities involves the regulation of different factors during successions. These findings add to our understanding of the forces that shape fungal community assembly, as well as the link between microbiome composition and stability over succession.

## Supplementary Information

Below is the link to the electronic supplementary material.

Supplementary file1 (PDF 910 KB)

## Data Availability

Raw sequences have been deposited in SRA (Bioproject: PRJNA925304).
