# Supplementary material for: Community composition of phytopathogenic fungi significantly influences ectomycorrhizal fungal communities during subtropical forest succession
Source: Appl Microbiol Biotechnol. 2024 Jan 10;108:99. doi: 10.1007/s00253-023-12992-5 (PMC10781812; doi:10.1007/s00253-023-12992-5)
Supplement: Supplementary file 1 [file 253_2023_12992_MOESM1_ESM.pdf]

Journal name: Applied Microbiology and Biotechnology

Title: Community composition of phytopathogenic fungi significantly influence ectomycorrhizal fungal communities during subtropical forest succession

Meirong Chen [1,2], Jiazhi Yang[3], Chunquan Xue[3]*, Tieyao Tu[1], Zhiyao Su[4], Hanhua Feng[3], Miaomiao Shi[1], Gui Zeng[5], Dianxiang Zhang[1]*, Xin Qian[6]*

[1] Key Laboratory of Plant Resources Conservation and Sustainable Utilization, South China Botanical Garden, Chinese Academy of Sciences, Guangzhou, China

2 University of Chinese Academy of Sciences, Beijing, China

3 Guangdong Forestry Survey and Planning Institute, Guangzhou, China

4 South China Agriculture University, Guangzhou, China

5 College of Life Sciences, China West Normal University, Nanchong, China

6 College of Life Sciences, Fujian Agriculture and Forestry University, Fuzhou, Fujian, China

***Correspondence**:

Xin Qian: E-mail: qxxb2006@163.com; Tel: 0086-20-15521102619

Chunquan Xue: E-mail: 2226043870@qq.com; Tel: 0086-20-87035590

Dianxiang Zhang: E-mail: dx-zhang@scbg.ac.cn; Tel: 0086-20-37252543

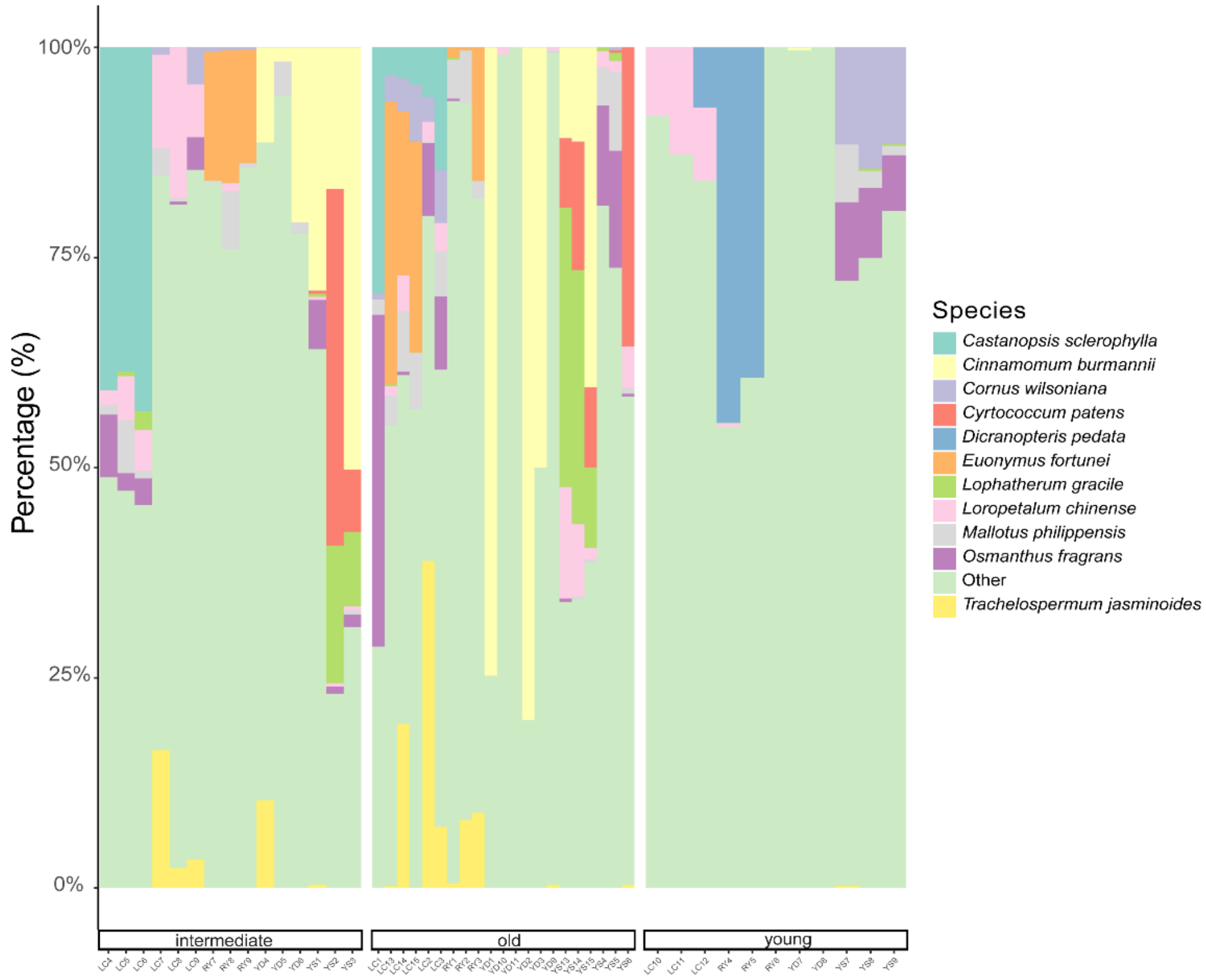

Fig. S1. Plant community taxonomic composition in secondary subtropical forests. The results are shown at the species level for all sample sites. The abundance of plants in the top 11 is shown, and all other species are grouped into "Other".

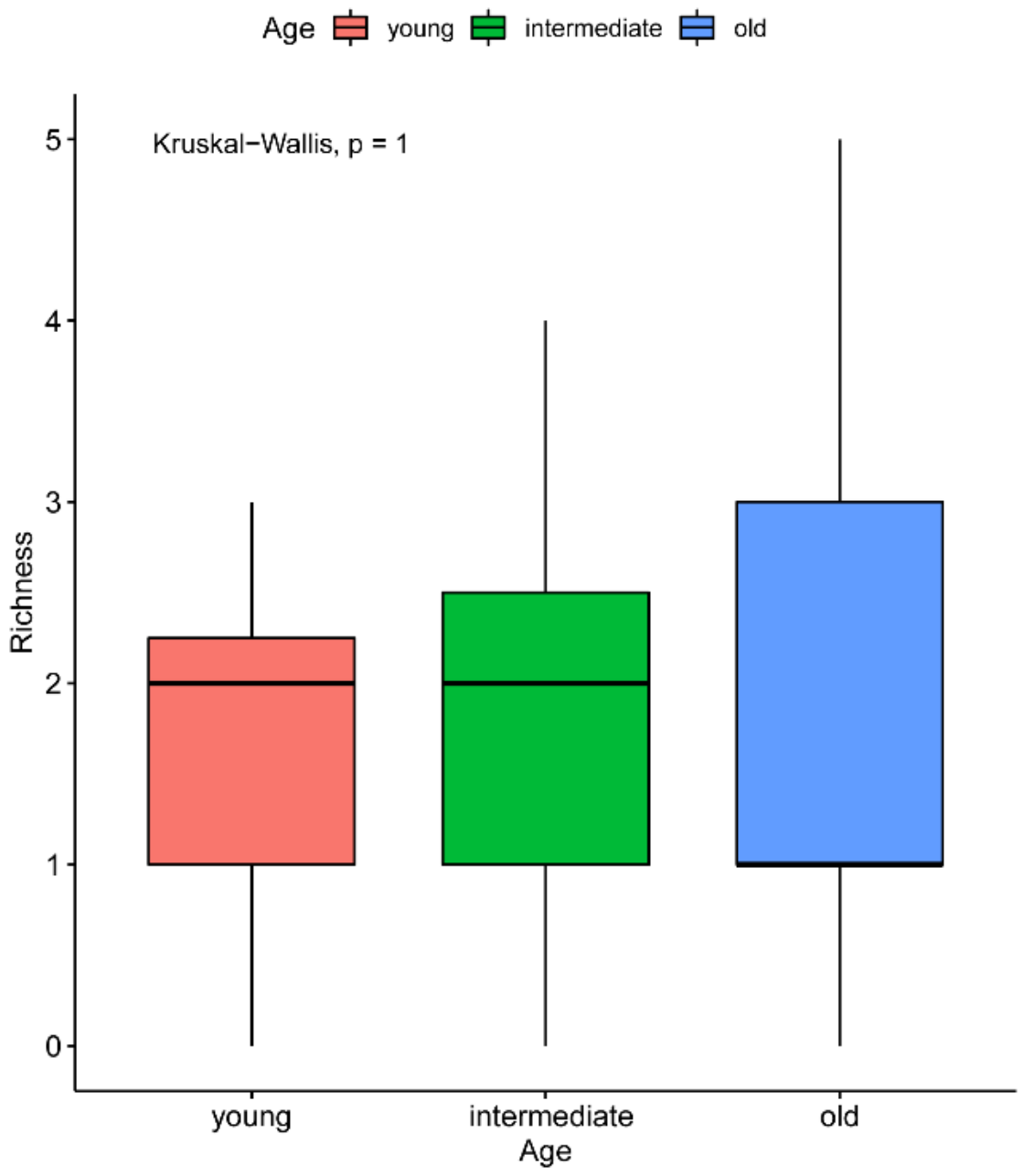

Fig. S2. EMF host plant richness of different sample sites in different age forests. There was no significant difference in EMF host plant richness among different stages (*P*=1) according to Kruskal-Wallis tests.

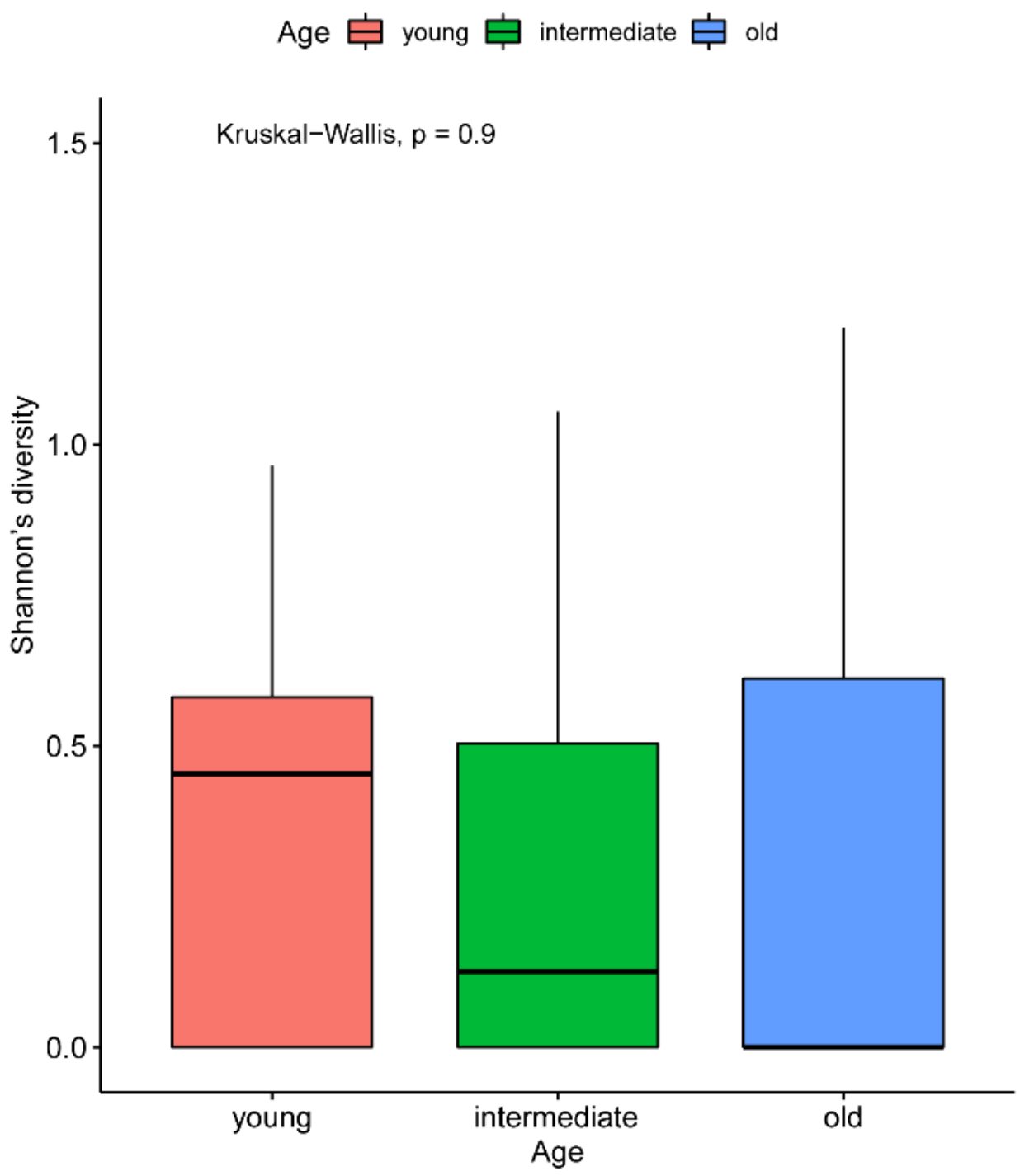

Fig. S3. EMF host plant Shannon diversity of different sample sites in different age forests. There was no significant difference in EMF host plant Shannon diversity among different stages (P=0.9) according to Kruskal-Wallis tests.

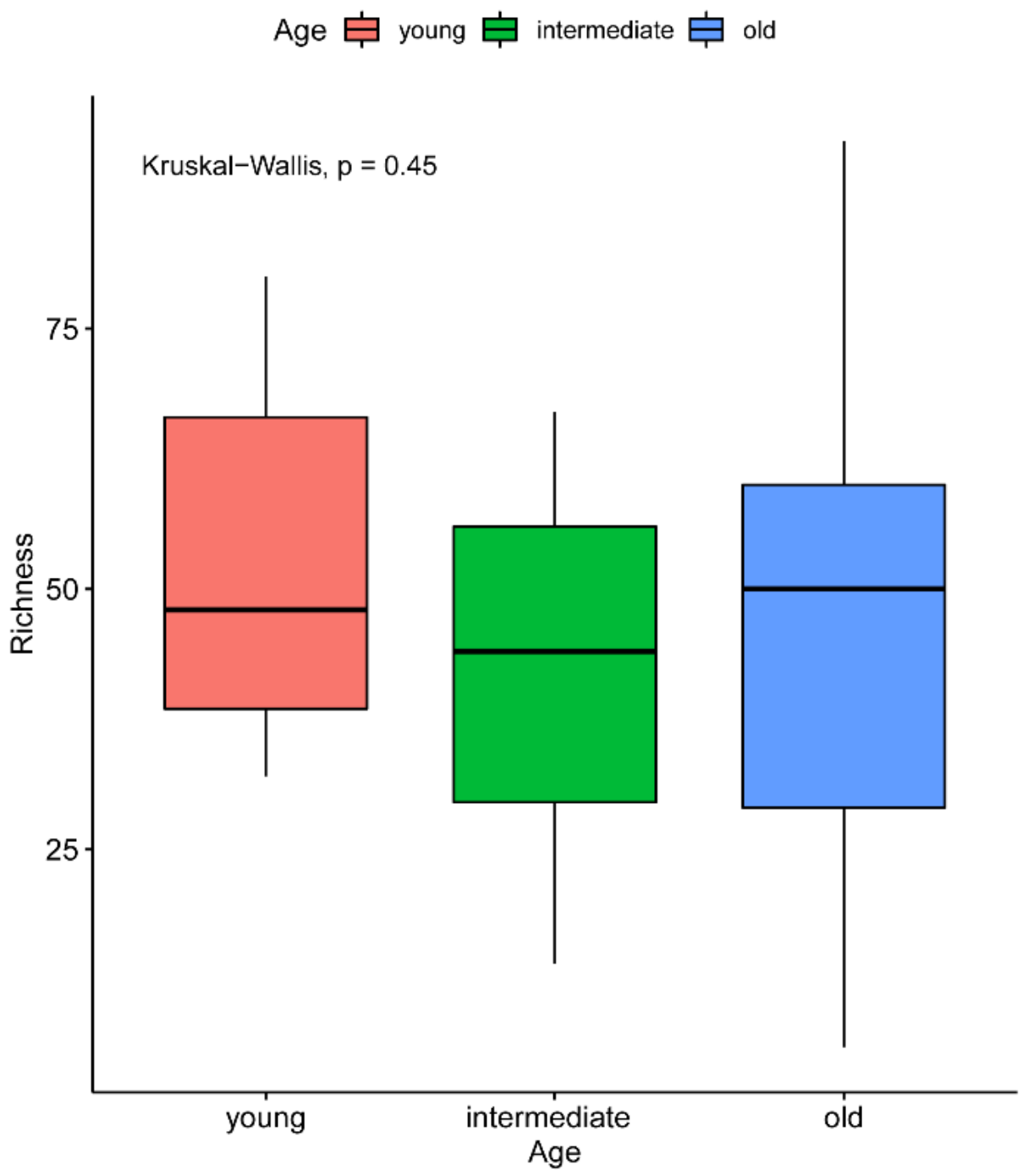

Fig. S4. All plant richness of different sample sites in different age forests. There was not significant difference in all plant richness over successions (*P*=0.45) according to Kruskal-Wallis tests.

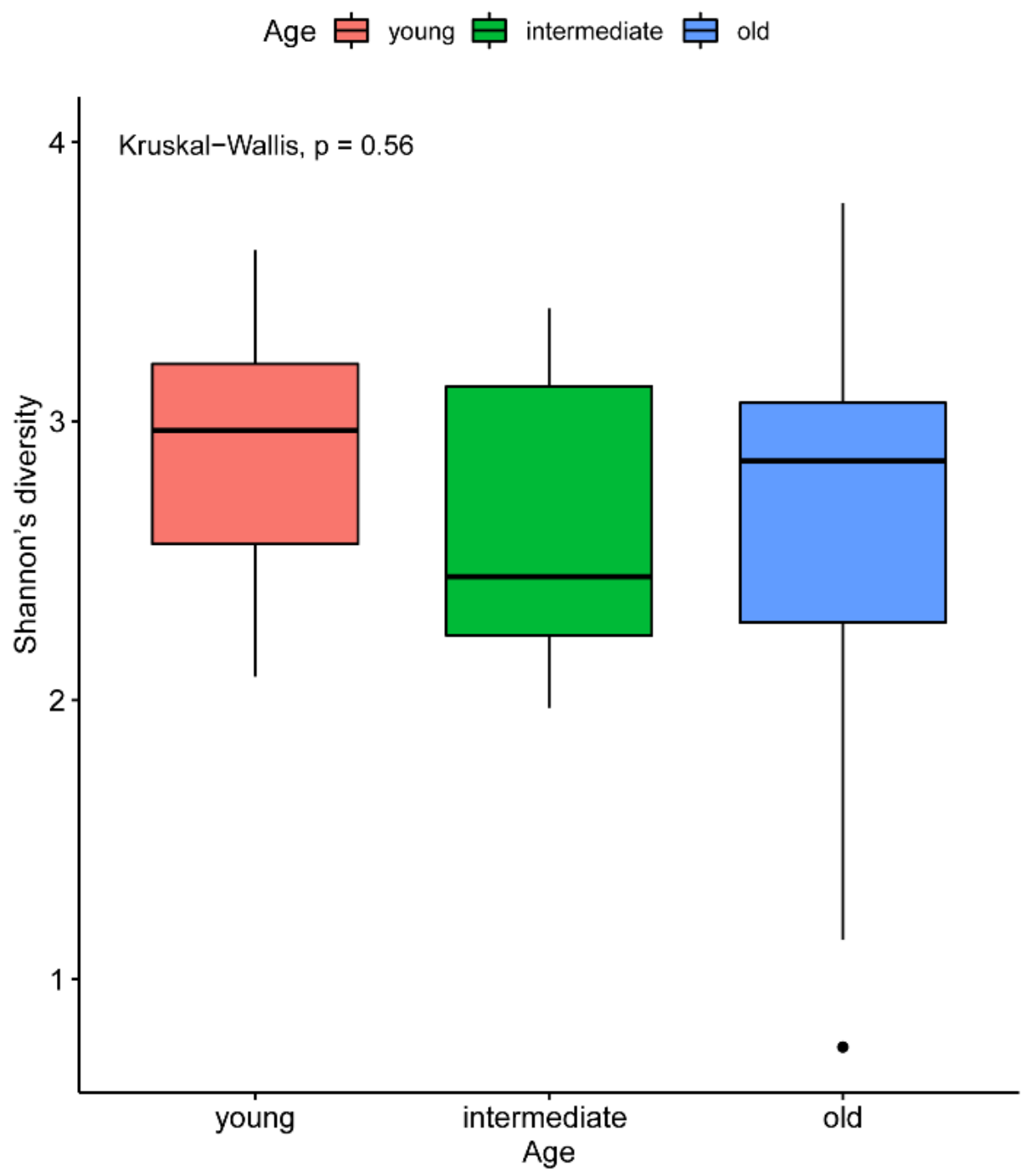

Fig. S5. All plant Shannon's diversity of different sample sites in different age forests. There was not significant difference in all plant Shannon's diversity over successions (*P*=0.56) according to Kruskal-Wallis tests.

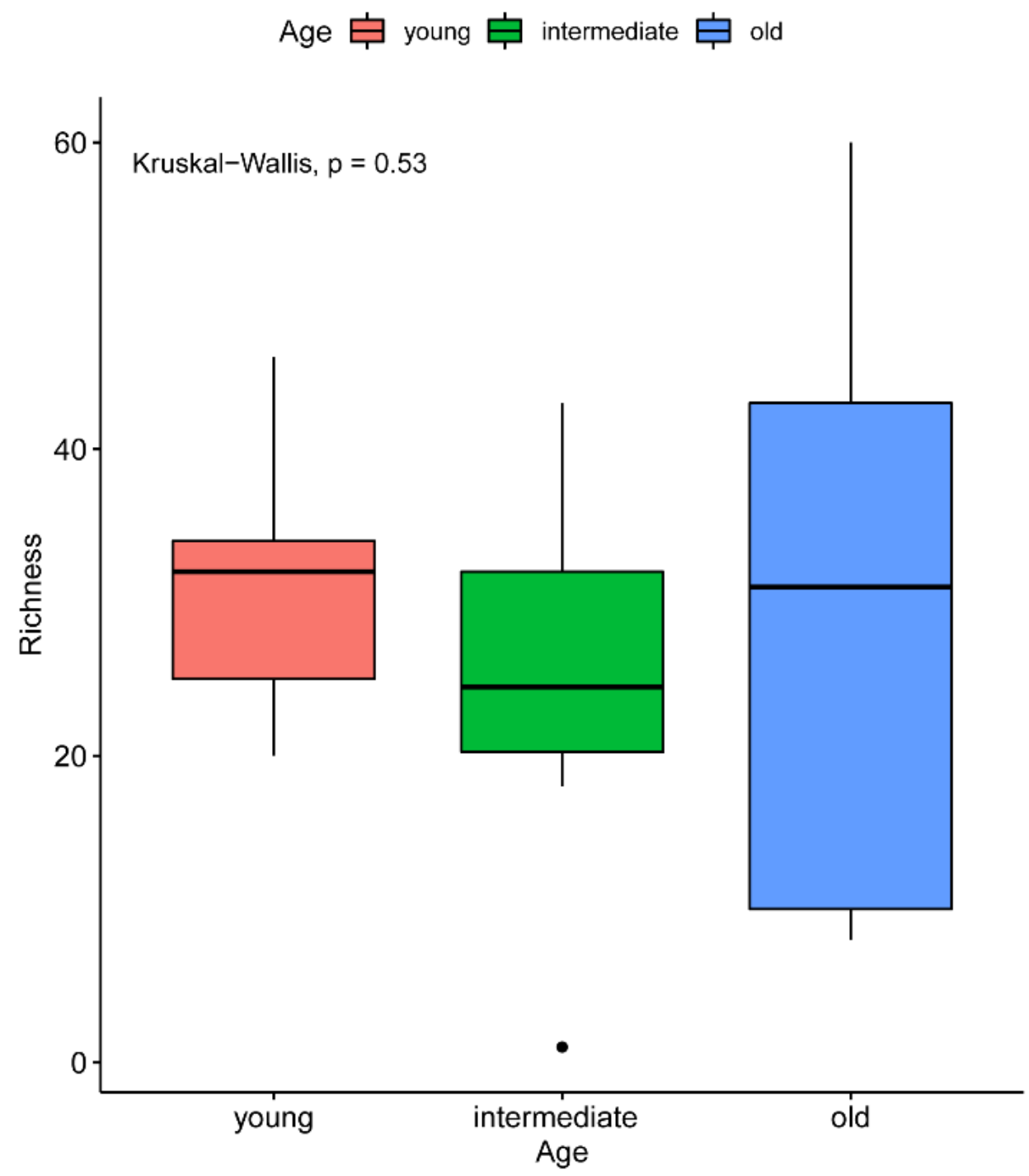

Fig. S6. Associated plant richness of different sample sites in different age forests. There was not significant difference in plant richness over successions (*P*=0.53) according to Kruskal-Wallis tests.

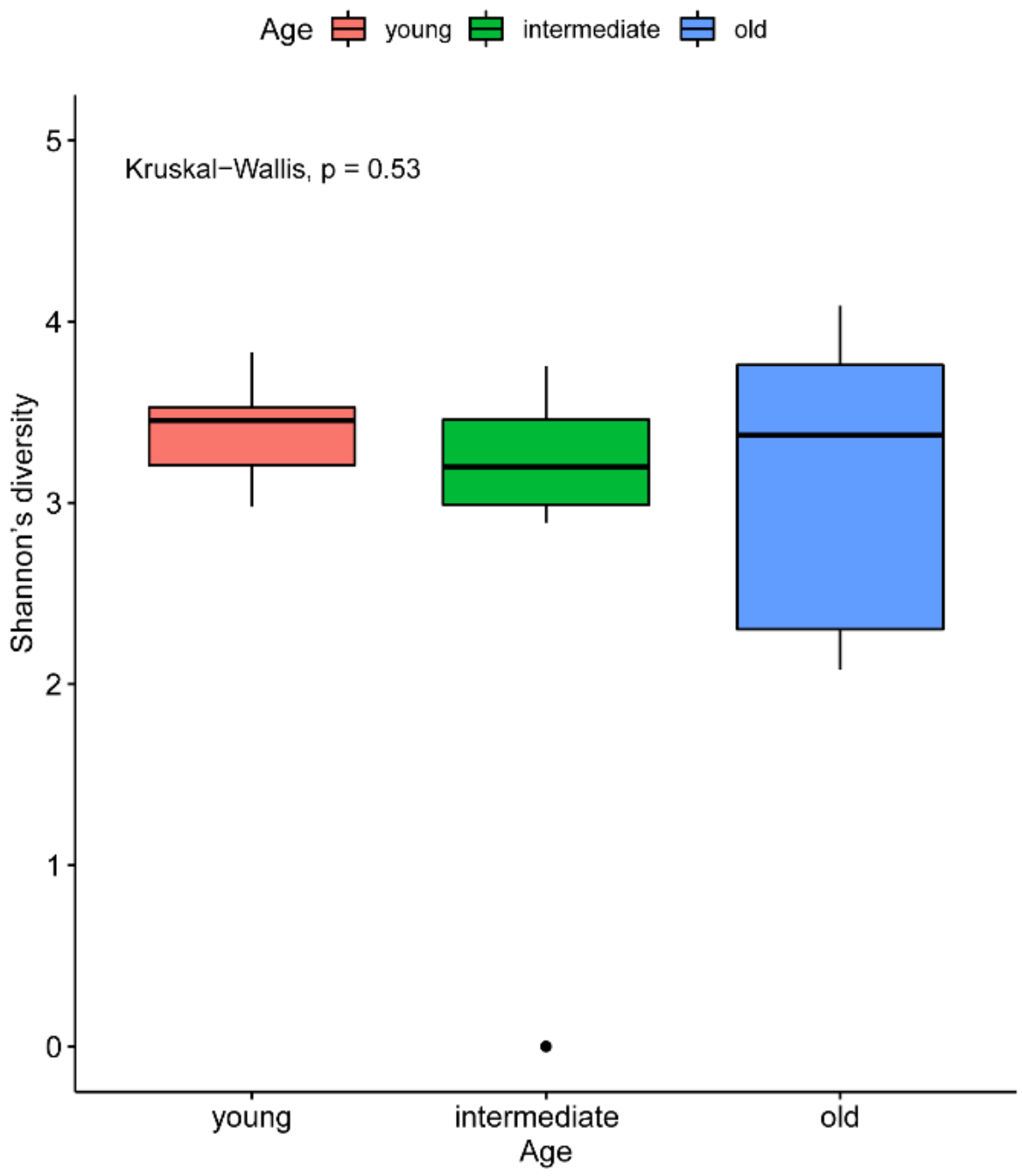

Fig. S7. Associated plant Shannon's diversity of different sample sites in different age forests. There was no significant difference in associated Shannon's diversity over successions (P=0.53) according to Kruskal-Wallis tests.

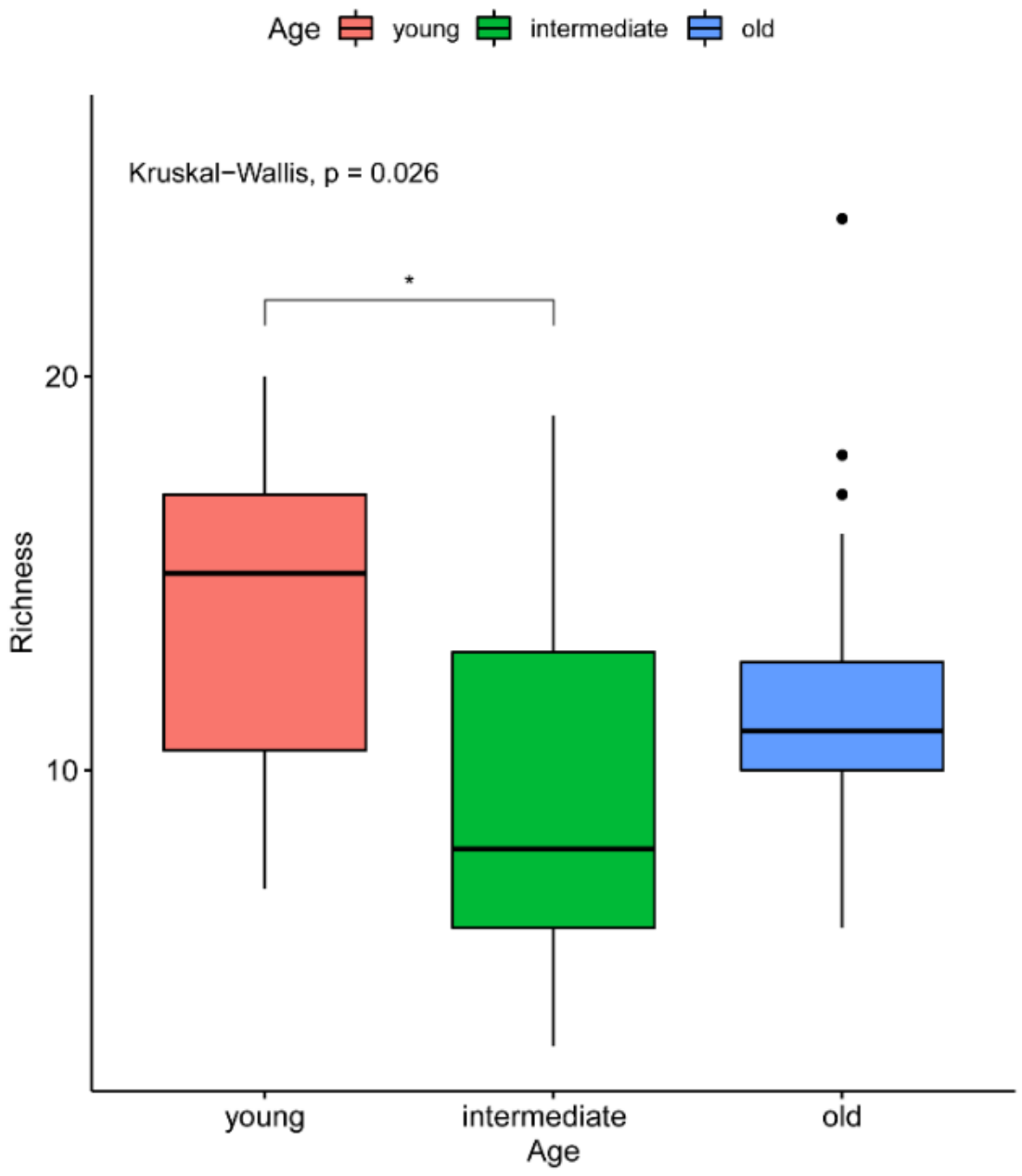

Fig. S8. Herb richness diversity of different sample sites in different age forests. The asterisk represents the significant difference in herb richness between young and intermediate forest (P=0.026) according to Kruskal-Wallis tests.

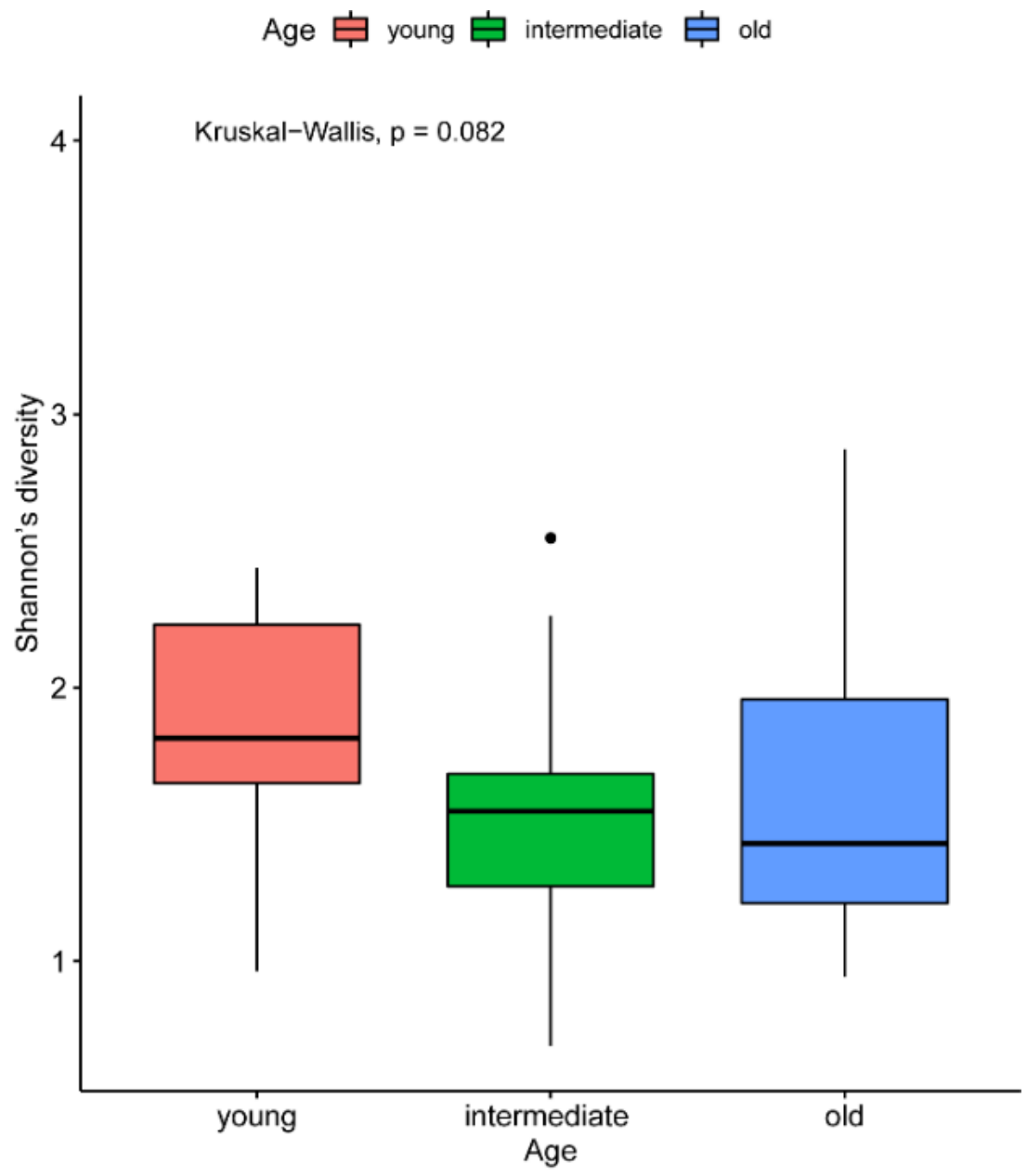

Fig. S9. Herb Shannon's diversity of different sample sites in different age forests. There was no significant difference in herb Shannon's diversity over successions (*P*=0.082) according to Kruskal-Wallis tests.

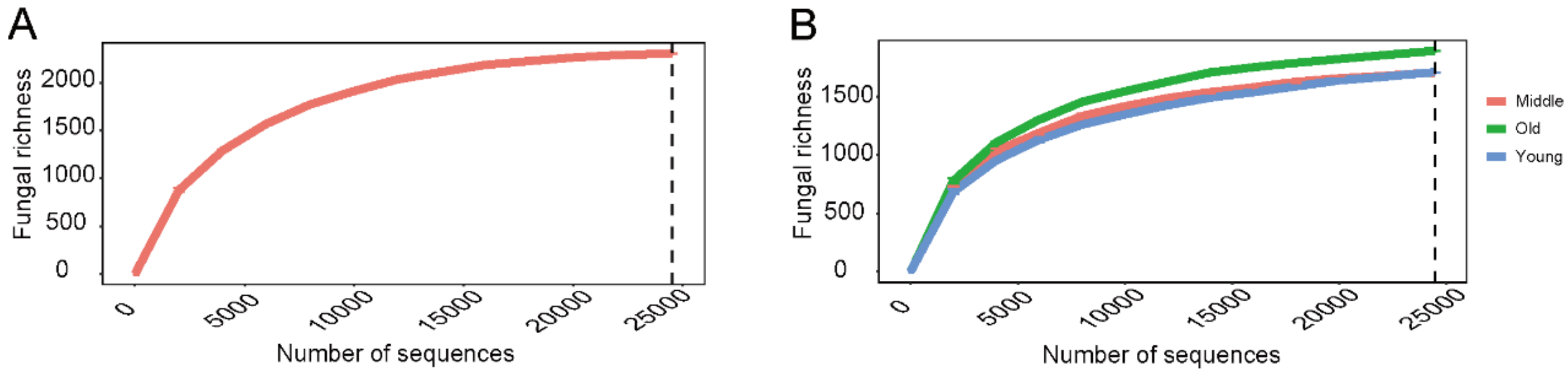

Fig. S10. (A) Rarefaction curve of fungal OTU in all samples. (B) Rarefaction curves of fungal OTU in different age forests.

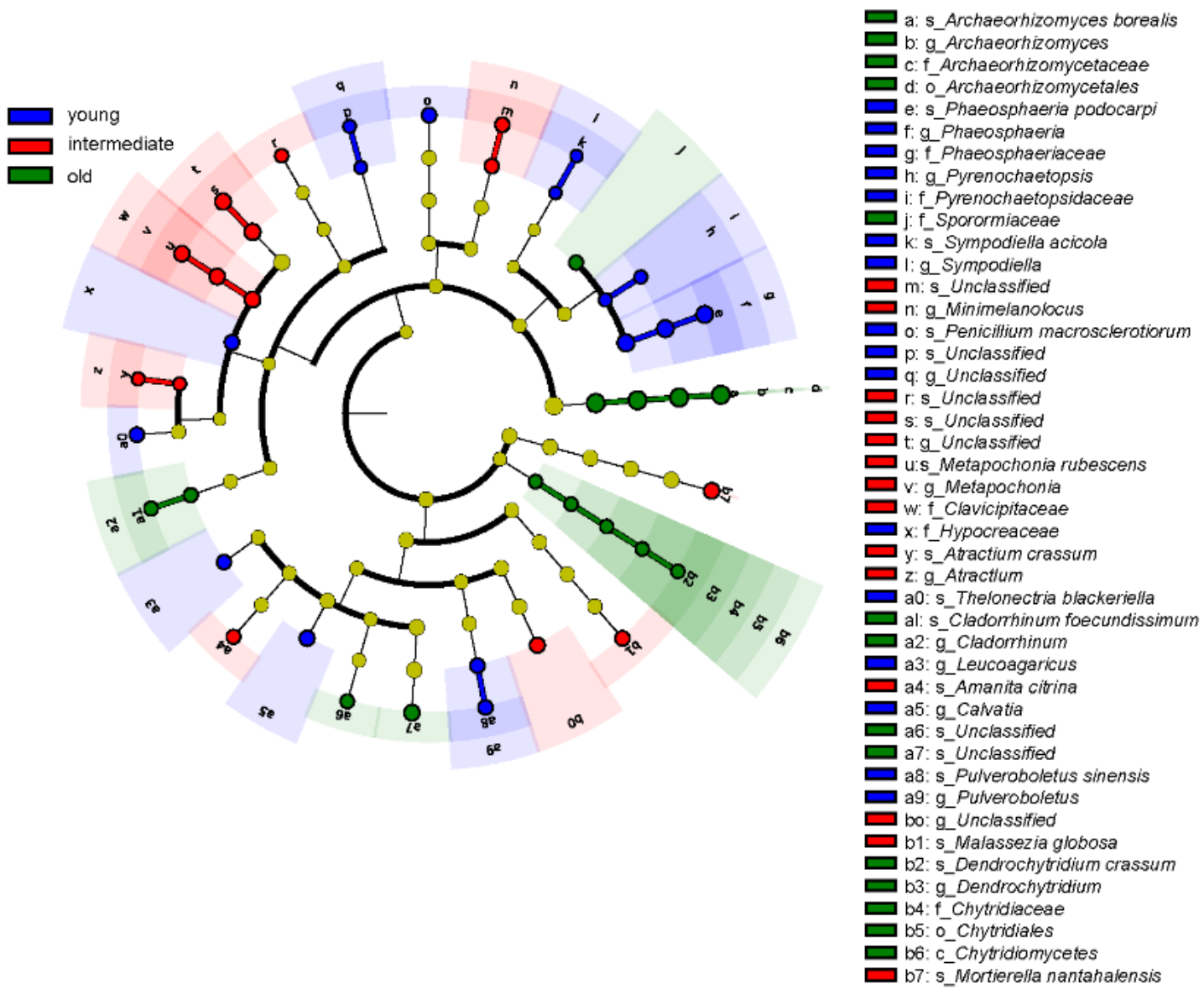

Fig. S11. Significant biomarkers among different successional stages were showed by Linear Discriminant Analysis Effect Size (LEfSe) using the Linear Discriminant Analysis (LDA) principle, only OTUs with a LDA score greater than 3.0 are displayed.

Table S1 Location and grouping of 48 sample sites

Sample sites	Longitude	Latitude	Slope	Elevation	Grouping
**LC1**	N25°12′58.02″	E113°04′02.04″	15	339	old
**LC2**	N25°12′57.58″	E113°04′03.48″	15	315	old
**LC3**	N25°12′57.58″	E113°04′03.48″	15	315	old
**LC4**	N25°12′57.58″	E113°04′03.48″	5	315	middle
**LC5**	N25°12′57.58″	E113°04′03.48″	10	315	middle
**LC6**	N25°12′57.58″	E113°04′03.48″	10	315	middle
**LC7**	N25°08′9.07″	E113°02′10.80″	5	473	middle
**LC8**	N25°08′8.92″	E113°02′11.05″	5	475	middle
**LC9**	N25°08′8.27″	E113°02′11.91″	5	476	middle
**LC10**	N25°07′58.72″	E113°01′42.96″	10	453	young
**LC11**	N25°07′58.60″	E113°01′42.91″	10	453	young
**LC12**	N25°07′58.62″	E113°01′43.49″	10	455	young
**LC13**	N25°09′50.38″	E113°05′03.98″	35	555	old
**LC14**	N25°09′49.77″	E113°05′03.20″	30	560	old
**LC15**	N25°09′49.83″	E113°05′03.46″	50	568	old
**RY1**	N25°05′59.70″	E113°10′06.93″	20	722	old
**RY2**	N25°06′00.77″	E113°10′07.08″	20	718	old
**RY3**	N25°05′59.70″	E113°10′07.09″	20	718	old
**RY4**	N25°00′09.32″	E113°07′25.23″	29	747	young
**RY5**	N25°00′07.97″	E113°07′24.97″	20	747	young
**RY6**	N25°00′08.15″	E113°07′25.04″	20	751	young
**RY7**	N25°00′08.13″	E113°07′25.01″	10	741	middle
**RY8**	N25°00′08.13″	E113°07′25.01″	10	741	middle
**RY9**	N25°06′45.51″	E113°09′33.14″	10	710	middle
**YD1**	N24°09′34.83″	E113°24′01.67″	5	79	old
**YD2**	N24°09′35.8455″	E113°23′58.32″	5	90	old
**YD3**	N24°09′35.8455″	E113°23′58.32″	5	90	old
**YD4**	N24°22′58.49″	E113°23′11.72″	5	113	middle
**YD5**	N24°22′58.13″	E113°23′13.77″	30	92	middle
**YD6**	N24°22′58.38″	E113°23′12.05″	30	92	middle
**YD7**	N24°18′21.09″	E112°47′12.59″	5	433	young
**YD7y**	N24°18′21.09″	E112°47′12.59″	5	433	young
**YD8**	N24°18′21.27″	E112°47′12.59″	5	433	young
**YD9**	N24°18′22.14″	E112°47′12.62″	25	437	old
**YD10**	N24°18′23.42″	E112°47′12.47″	30	436	old
**YD11**	N24°18′23.42″	E112°47′12.47″	30	436	old
**YS1**	N24°29′14.81″	E112°38′32.58″	12.5	132	middle
**YS2**	N24°29′14.81″	E112°38′32.58″	12.5	132	middle
**YS3**	N24°29′14.81″	E112°38′32.58″	12.5	132	middle
**YS4**	N24°32′26.82″	E112°43′30.71″	35	344	old
**YS5**	N24°32′26.82″	E112°43′30.71″	35	344	old
**YS6**	N24°32′26.82″	E112°43′30.71″	35	344	old
**YS7**	N24°36′42.37″	E112°43′30.73″	30	517	young
**YS8**	N24°36′42.37″	E112°43′30.73″	30	517	young
**YS9**	N24°36′42.37″	E112°43′30.73″	30	517	young
**YS13**	N24°29′17.00″	E112°38′24.66″	19	123	old
**YS14**	N24°29′17.88″	E112°38′24.2″	20	140	old
**YS15**	N24°29′18.95″	E112°38′23.21″	5	164	old

Table S2 Information of EMF host plant species in each sample site

Sample sites	QF	CM	PO	TT	CT	CS1	TW	CH	QA	CF1	CS2	CF2	CL1	CF3	PM	CG	CF4	FL	CL2	PE	CS3	AC	TF	RT
LC1	0	0	0	0	0	0	0	0	0	0	114	0	0	0	0	0	0	0	0	0	0	0	0	0
LC2	0	0	0	0	0	0	0	0	0	0	30	0	0	0	0	0	0	0	0	0	0	0	0	0
LC3	0	0	0	0	0	0	0	0	0	0	30	0	0	0	0	0	0	0	0	0	0	0	0	0
LC4	0	0	0	0	0	0	0	0	0	0	71	0	0	0	2	0	0	0	0	0	0	0	0	0
LC5	1	0	0	0	0	0	0	0	0	0	74	0	0	0	9	1	0	0	0	0	0	0	0	0
LC6	0	0	0	0	0	0	0	0	0	0	136	0	0	0	11	0	0	0	0	0	0	0	0	0
LC7	13	0	0	0	0	0	0	0	4	0	0	0	0	0	0	0	0	0	0	0	0	0	0	0
LC8	15	0	0	0	0	0	0	0	0	0	0	0	0	0	1	0	0	0	1	0	0	0	1	0
LC9	8	0	0	0	0	0	0	0	0	0	0	0	0	0	0	0	0	0	0	0	0	0	0	0
LC10	0	0	0	0	0	0	0	0	1	0	0	0	0	0	1	0	0	0	0	11	0	0	0	0
LC11	0	0	17	0	0	0	0	0	0	0	0	0	0	0	0	0	0	0	0	18	0	0	0	0
LC12	0	0	0	0	0	0	0	0	0	0	0	0	0	0	0	0	0	0	0	9	0	0	0	0
LC13	3	0	0	0	0	0	0	0	0	1	15	0	10	0	0	0	0	0	0	0	0	0	0	0
LC14	9	0	0	0	0	0	0	0	1	0	19	0	2	0	4	0	0	0	0	0	0	0	0	0
LC15	4	0	0	0	0	0	0	0	0	0	18	0	0	0	3	0	0	0	0	0	0	0	4	0
RY1	0	0	0	2	0	52	0	0	0	0	0	0	0	0	0	0	2	12	0	0	0	0	0	0
RY2	0	0	0	0	0	14	0	0	0	0	0	0	0	0	0	0	0	0	0	0	1	0	1	0
RY3	0	0	0	2	0	72	0	0	0	0	0	0	0	0	1	0	9	0	0	2	0	0	0	0
RY4	0	0	0	0	0	0	0	0	0	0	0	0	0	0	0	0	0	0	0	0	0	0	0	0
RY5	0	0	0	0	0	0	0	2	0	0	0	0	0	0	0	0	0	0	0	4	0	0	0	0
RY6	0	0	0	0	0	0	0	0	0	0	0	0	0	0	0	0	0	0	2	0	0	0	0	0
RY7	0	0	0	0	1	0	2	0	0	0	0	0	0	2	0	0	0	0	0	0	0	0	0	0
RY8	0	0	0	0	0	0	3	0	0	0	0	0	0	0	0	15	0	0	0	0	0	0	0	0
RY9	0	0	0	0	0	0	1	0	0	0	0	0	0	87	0	60	0	0	0	0	0	0	1	0
YD1	0	0	0	0	0	0	0	0	0	0	0	0	0	0	1	0	0	0	0	0	0	0	0	0
YD2	0	0	0	0	0	0	0	0	0	0	0	0	0	0	0	0	0	0	0	0	0	0	0	0

YD4	0	0	0	0	0	0	0	0	0	0	0	0	0	0	0	0	0	0	0	0	0	0	0	0
YD5	0	0	0	0	0	0	0	0	0	0	0	0	0	0	0	0	0	0	0	0	0	0	0	0
YD6	0	0	0	0	0	0	0	0	0	0	0	0	0	0	0	0	0	0	0	0	0	0	0	0
YD7y	0	0	0	0	0	0	0	0	0	0	0	0	0	0	0	0	0	0	3	0	0	0	0	0
YD3	0	0	0	0	0	0	0	0	0	0	0	0	0	0	2	0	0	0	0	0	0	6	0	0
YD7	0	0	0	0	0	0	0	0	0	0	0	0	0	0	0	0	0	0	3	0	0	0	0	0
YD8	0	0	0	0	0	0	0	0	0	0	0	0	0	0	0	0	0	0	0	0	0	0	0	0
YD9	0	0	0	0	0	0	0	0	0	0	0	0	0	0	27	0	0	0	1	0	0	0	3	0
YD10	0	0	0	0	0	0	0	0	0	0	0	2	0	0	36	0	0	0	0	0	0	0	0	0
YD11	0	0	0	0	0	0	0	0	0	0	0	0	0	0	47	0	0	0	0	1	0	0	0	0
YS1	0	0	0	0	0	0	0	0	0	0	0	0	0	0	7	0	0	0	0	0	0	0	0	0
YS2	0	0	0	0	0	0	0	0	0	0	0	0	0	0	18	0	0	0	0	0	0	0	0	0
YS3	0	0	0	0	0	0	0	0	0	0	0	0	0	0	13	0	0	0	0	0	0	0	0	0
YS4	0	0	0	0	0	0	0	0	0	0	0	0	0	0	0	9	0	0	0	0	0	0	0	0
YS5	0	0	0	0	0	0	0	0	0	0	0	0	0	0	0	0	0	0	0	0	0	0	0	0
YS6	0	2	0	0	0	0	0	0	0	0	0	0	0	0	0	6	0	0	3	0	0	0	0	0
YS7	0	5	0	0	0	0	0	0	0	0	0	0	0	0	0	1	0	0	0	0	0	0	0	0
YS8	0	0	0	0	0	0	0	0	0	0	0	0	0	0	0	0	0	0	0	0	0	0	6	0
YS9	0	1	0	0	0	0	0	0	0	0	0	0	0	0	0	0	0	0	4	0	0	0	4	0
YS13	0	0	0	0	0	0	0	0	0	0	0	0	0	0	19	0	0	0	0	0	0	0	0	0
YS14	0	0	0	0	0	0	0	0	0	0	0	0	0	0	14	0	0	0	0	0	0	0	0	1
YS15	0	0	0	0	0	0	0	0	0	0	0	0	0	0	16	0	0	0	0	0	0	0	0	0

QF: *Quercus fabri*; CM: *Castanea mollissima*; PO: *Platycladus orientalis*; TT: *Tilia tuan*; CT: *Carpinus turczaninowii*; CS1: *Cyclobalanopsis stewardiana*; TW: *Taxus wallichiana*; CH: *Castanopsis hystrix*; QA: *Quercus aliena*; CF1: *Castanopsis fargesii*; CS2: *Castanopsis sclerophylla*; CF2: *Castanopsis fissa*; CL1: *Castanopsis lamontii*; CF3: *Castanopsis faberi*; PM: *Pinus massoniana*; CG: *Cyclobalanopsis glauca*; CF4: *Cephalotaxus fortune*; FL: *Fagus longipetiolata*; CL2: *Cunninghamia lanceolate*; PE: *Pinus elliottii*; CS3: *Castanea* sp.; AC: *Acacia confuse*; TF: *Trachycarpus fortune*; RT: *Rhodomyrtus tomentosa*

Table S3 Information of plant and soil factors for each sample site

	Plant factors							Soil factors																	
**Sample sites**	**HSD**	**APSD**	**TSD**	**ECMTSD**	**PSD**	**W**	**AW**	**WC**	**LF**	**HS**	**pH**	**OM**	**TN**	**TP**	**TK**	**N**	**P**	**K**	**Ca**	**Mg**	**Cu**	**Zn**	**Fe**	**Mn**	**B**
**LC1**	2.96	3.08	1.87	0	3.5	2.54	2.03	0.21	1.2	3.5	6.61	16.22	1.1	0.34	9.18	70.55	0.21	32.38	945.87	44.2	0.57	0.29	31.46	71.99	0.05
**LC2**	1.92	1	2.68	0	3.71	4.1	1.33	0.17	3.16	3.64	6.78	16.16	1.14	0.3	11.98	49.84	0.11	43.27	2386.5	26.65	0.61	0.31	12.57	41.9	0.04
**LC3**	3.02	1	2.52	0	3.14	4.31	1.42	0.17	2.58	3.58	6.73	13.77	1.06	0.28	11.68	49.19	0.11	35.38	1546.2	26.18	0.34	0.1	13.58	28.55	0.04
**LC4**	0.66	3.89	2.74	0.13	3.26	3.82	0.2	0.17	4.06	2.86	6.57	15.44	1.1	0.31	11.12	56.31	0.21	29.13	1034.4	72.48	0.44	0.22	24.71	87.94	0.06
**LC5**	1.17	1	2.58	0.46	3.14	4.18	1.66	0.17	2.6	4.3	6.43	12.97	0.8	0.32	7.48	44.01	0.3	22.38	595.05	41.72	0.46	0.33	19.23	64.69	0.07
**LC6**	1.64	3.93	2.44	0.27	3.14	3.74	0.85	0.18	2.6	4.2	6.3	14.15	0.89	0.35	7.66	47.25	0.3	23.36	664.04	60.4	0.29	0.14	18.42	47.57	0.08
**LC7**	1.53	4.18	2.96	0.55	3.71	3.85	1.08	0.13	1.6	2.06	6.01	18.99	1.12	0.2	8.43	75.73	0.3	26.34	771.4	28.31	0.42	0.22	35.99	71.23	0.04
**LC8**	2.5	4.47	2.54	0.63	3.33	3.37	1.11	0.15	1.2	2.12	6.17	18.07	1.14	0.24	8.2	72.49	0.4	31.6	1656.2	49.18	0.37	0.14	17	70.29	0.05
**LC9**	2.16	1	2.87	0	3.3	3.35	1.85	0.16	2.12	2.36	6.24	23.61	1.39	0.23	8.41	99.68	0.3	32	2152.4	44.69	0.49	0.22	17.21	48.38	0.03
**LC10**	1.55	3.45	2.08	0.54	2.89	3.53	0.89	0.13	2.68	2.64	6.36	18.01	0.95	0.12	7.28	56.96	0.49	20.3	834.09	13.82	0.44	0.13	24.91	35.59	0.07
**LC11**	1.82	2.98	2.46	0.69	2.77	2.47	1.57	0.15	1.98	2.4	6.03	18.22	0.99	0.11	9.88	60.2	0.3	22.83	702.57	10.74	0.29	0.1	24.3	17.28	0.08
**LC12**	2.44	3.12	2.03	0	2.4	2.36	1.12	0.18	2.26	3.5	6.01	15.34	1	0.11	13.34	48.55	0.21	21.03	670.24	12.71	0.18	0.07	14.58	3.84	0.08
**LC13**	2.1	4.76	3.14	1.06	3.81	6.84	1.99	0.19	1.34	5.14	5.7	22.61	1.77	0.32	20.46	95.8	0.11	20.25	1694	14.95	1.19	0.33	32.07	45.79	0.14
**LC14**	2.26	3.08	3.04	1.19	3.85	11.33	2.11	0.19	2.96	2.64	5.57	26.77	1.76	0.27	18.33	102.92	0.4	29.29	1906.5	20.61	1.43	0.33	61.1	21.08	0.09
**LC15**	2.47	3.77	2.8	1.08	3.56	5.54	2.23	0.17	2.12	5.2	5.62	38.06	2.49	0.32	20.07	173.47	0.59	32.98	2705.3	27.91	1.5	0.39	50.34	49.81	0.1
**RY1**	3.06	4.37	3.45	0.72	3.99	7.75	1.62	0.17	2.86	4.26	5.6	33.37	1.9	0.38	15.88	139.81	1.17	35.05	1838.5	31.42	0.38	0.47	58.56	55.61	0.12
**RY2**	2.38	1	3.32	0.46	3.91	10.76	2.2	0.15	1.9	3.36	5.66	32.43	1.97	0.44	14.64	148.87	1.07	37.22	2637	35.1	0.21	0.62	36.4	52.78	0.1
**RY3**	2.4	3.3	3.38	0.61	4.08	9.3	1.59	0.15	1.36	2.48	5.53	26.89	1.59	0.21	22.1	121.69	0.97	34.52	1278.5	31.06	0.21	0.47	51.39	13.15	0.12
**RY4**	2.3	3.5	2.01	0	2.71	0.14	0.36	0.15	1.12	2.54	5.46	14.58	0.81	0.1	8.23	48.55	0.69	15.09	83.09	7.31	0.24	0.28	16.8	23.34	0.1
**RY5**	0.96	3.3	1.47	0.64	2.2	0.04	5.32	0.18	1	5.82	5.29	26.24	1.41	0.14	11.05	94.5	0.4	21.07	82.32	10.48	0.54	0.68	26.14	30.1	0.1
**RY6**	1.53	3.83	2.58	0	3	0.08	10.79	0.16	0.68	5.68	5.46	17.69	1.2	0.13	11.54	74.44	0.21	23.98	750.53	17.79	0.44	0.13	18.22	30.58	0.09
**RY7**	0.69	4.22	3.05	1.05	3.61	6.47	1.45	0.15	1.5	3.8	5.67	28.2	1.54	0.16	8.97	108.09	0.58	29.78	1782.22	28.64	0.63	0.4	33.63	54.64	0.13
**RY8**	1.62	1	3.05	0.45	3.71	11.6	0.62	0.12	0.5	4.1	5.72	32.52	1.75	0.15	10.23	121.04	0.78	34.69	739.06	22.5	0.57	0.19	46.93	5.23	0.1
**RY9**	1.77	4.14	3.11	0.75	3.61	7.61	0.68	0.09	0.9	2.15	5.61	19.22	1.19	0.16	4.39	70.55	6.8	12.23	133	5.16	0.22	0.86	55.64	1.88	0.03
**YD1**	1	3.2	0.66	0	1.95	26.29	1	0.15	0.2	2	6.94	28.43	1.68	0.54	18.34	101.62	0.78	36.01	1779.6	38.58	1.74	3.21	39.5	125.5	0.14

**YD2**	1	1	0.76	0	1.79	5.51	1	0.16	0.24	2.8	7.06	21.73	1.5	0.4	17.96	63.43	0.21	45.77	1932.7	47.48	0.87	1.29	17	98.52	0.11
**YD3**	1	1	1.37	0.56	2.3	10.09	1	0.16	0.4	3	6.95	19.2	1.6	0.35	27.28	82.85	0.21	36.29	1507.9	30.31	0.61	1.2	17.21	81.5	0.15
**YD4**	1.67	1	1.68	0	2.3	0.46	1.61	0.19	1	1.3	6.96	16.58	1.08	0.64	18.71	67.32	5.87	36.6	2808.8	90.78	1.57	1.02	25.53	33.65	0.12
**YD5**	1.7	1	2.06	0	2.89	0.08	0.44	0.26	1.2	1.5	7.1	28.58	1.73	0.9	12.99	111.33	2.13	43.96	2271.5	156.45	2.17	2.82	28.59	85.89	0.12
**YD6**	1.33	1	2.41	0	3.09	7.4	1.3	0.23	0.9	1.5	6.81	30.92	1.76	0.66	16.92	128.16	6.83	41.99	2075.3	40.15	2.49	3.19	51.18	41.74	0.14
**YD7**	1.78	3.81	1	0	1.95	0.26	0.44	0.15	0.5	0.2	6.99	18.67	1.13	0.4	17.58	74.44	0.88	28.79	1216.1	9.18	0.96	1.24	14.99	49.22	0.09
**YD7y**	1.78	3.81	1	0	1.95	0.26	0.44	0.18	0.4	0.2	7.02	19.97	1.23	0.39	20.51	68.61	0.4	37.4	1640	17.1	0.77	0.54	14.58	47.29	0.09
**YD8**	1.75	3.04	1.07	0	1.39	0.05	0.76	0.18	0.2	0.1	7.04	16.13	1.14	0.36	22.05	66.02	0.3	43.34	1090.6	19.58	0.63	0.48	17.41	51.18	0.06
**YD9**	2.14	5.07	2.18	0.46	2.89	4.59	2.13	0.17	1.3	2	6.87	16.03	1.16	0.28	20.33	63.43	0.3	32.52	988.4	13.01	0.32	0.18	15.99	30.74	0.1
**YD10**	3.87	1	2.23	0.21	2.83	8.53	2.46	0.12	1.5	3	6.84	10.79	0.93	0.21	17.86	48.55	0.21	38.31	950.01	24.01	0.2	0.22	11.97	34.59	0.11
**YD11**	3.17	1	2.37	0.1	3	9.82	2.28	0.16	1.2	2.5	6.83	14.8	1.09	0.26	23.11	64.08	0.21	34.89	1005.5	14.86	0.24	0.09	14.99	17.65	0.07
**YS1**	1.22	4.75	0.77	0	1.79	8.39	1.48	0.13	1.18	0.76	5.41	17.9	1.78	0.24	11.03	47.9	0.2	37.02	2270.16	18.24	0.6	0.63	26.61	42.07	0.1
**YS2**	1	4.22	0.67	0	1.95	9.98	1.41	0.11	0.82	2.54	5.57	22.81	1.36	0.16	8.87	56.31	0.58	27.36	1890.46	20.74	0.98	0.54	38.47	22.58	0.08
**YS3**	1.55	4.58	0.42	0	1.79	6.03	0.91	0.12	1.08	1.34	5.64	24.73	1.75	0.21	11.02	74.44	0.39	34.14	2205.04	19.14	0.65	0.42	37.83	33.65	0.13
**YS4**	1.98	4.33	2.62	0	3.09	9.52	1.61	0.15	0.62	1.14	5.52	42.62	3.39	0.55	15.55	190.3	0.58	70.81	2677.96	79.26	1.7	2.43	47.63	95.09	0.17
**YS5**	2.39	4.71	2.58	0	3.4	3.52	1.89	0.17	0.92	0.54	5.65	41.58	3.12	0.47	15.41	183.18	0.3	64.38	2824.82	68.18	1.52	1.34	35.07	110.5	0.15
**YS6**	2.2	4.81	2.54	0.99	3.04	3.48	1.89	0.16	0.5	1.18	5.61	28.15	2.47	0.43	16.76	124.28	0.2	48.6	1194.66	50.36	0.89	0.69	43.01	71.17	0.14
**YS7**	2.18	3.5	3.05	0.45	3.76	4.35	0.49	0.12	1.84	3.92	5.93	34.36	2.83	0.47	22.85	141.75	0.2	50.12	3442.44	67	0.71	0.37	23.36	64.48	0.11
**YS8**	2.28	3.32	2.82	0	3.56	3.96	0.62	0.11	0.78	1.26	6.2	25.87	2.21	0.38	22.64	105.5	0.01	54.2	6015.42	50.96	0.56	0.24	19.7	42.12	0.05
**YS9**	1.96	3.56	2.95	0.96	3.71	5.47	1.1	0.14	1.32	3.88	6.37	30.75	2.51	0.45	23.66	119.74	0.11	65.75	2977.22	61.58	0.72	0.33	31.31	65.87	0.1
**YS13**	2.14	1	1.86	0	2.71	6.41	2.07	0.14	2.36	3.8	6.58	28.3	2.31	0.2	11.74	79.61	0.49	29.31	1474.76	30.96	0.87	0.52	32.42	29.74	0.17
**YS14**	2.51	4.82	1.58	0.24	2.48	7.97	12.05	0.16	3.1	3.64	6.45	20.7	1.95	0.2	14.78	57.61	0.11	32.63	799.66	22.34	0.5	0.23	23.99	2.59	0.12
**YS15**	2.78	4.66	0.93	0	2.3	11.55	1.84	0.14	2.78	4.18	5.81	23.06	1.58	0.22	10.14	66.67	0.39	19.85	513.7	12.54	0.75	0.32	36.71	1.3	0.11

HSD: Herb Shannon's diversity; APSD: Associated plant Shannon's diversity; TSD: Tree Shannon's diversity; ECMTSD: ECMtree Shannon's diversity; PSD: Plant Shannon's diversity; WC: Water content; LF: Litter fall; HS: Humic substances; OM: organic matter; W: tree biomass; AW: associated tree biomass; TN: Total nitrogen; TP: Total phosphorus; TK: Total potassium;

Table S4 Results of the partial mantel test for phytopathogenic fungal abundance to test the various factors effect during forest succession

Responder	Forest Age	Parameter	Coefficient	t	*P*	Sig.	Model parameters
Phytopathogenic fungi	Young	ECM tree Shannon's diversity	-0.65	-4.362	5.98E-05	***	R^2_{adj}=0.3215, F (6,53) =5.659, P=0.0001341
		Humic substances	1.23	4.116	0.000135	***	
		Organic matter	0.39	2.598	0.012109	*	
		Plant Shannon's diversity	-0.52	-2.28	0.026635	*	
		Quick acting K	0.67	4.142	0.000124	***	
		Slope	-0.71	-2.943	0.004812	**	
	Intermediate	Litterfall	-0.35	-2.565	0.01241	*	R^2_{adj}=0.3195, F (2,72) =9.684, P=2.719e-06
		Total N	-0.46	-3.337	0.00134	**	
	Old	Available B	0.17	1.749	0.0834		R^2_{adj}=0.06682, F (3,101) =3.482, P=2.886e-07
		Exchangeable Ca	0.22	2.181	0.0315	*	
		Slope	-0.21	-2.099	0.0383	*	

Table S5 Results of the partial mantel test for EMF abundance to test the various factors effect during forest succession

Responder	Forest age	Factors	Mantel statistic r	*P*
EMF	Young	Tree Shannon's diversity	0.1162	0.0200
		Available Fe	0.1266	0.0010
		Available Zn	0.1725	0.0010
		pH	0.0920	0.0290
		Litterfall	0.1666	0.0020
		Humic substance	0.1660	0.0020
		Water content	0.1166	0.0080
		Slope	0.1589	0.0010
	Intermediate	Herb Shannon's diversity	0.1380	0.0010
		Associated tree Shannon's diversity	0.1199	0.0010
		Tree Shannon's diversity	0.2201	0.0010
		Plant Shannon's diversity	0.1624	0.0010
		Tree weight	0.0987	0.0240
		Available B	0.0975	0.0040
		Available N	0.0620	0.0220
		Total P	0.0757	0.0450
		Total N	0.0688	0.0220
		Organic matter	0.0609	0.0350
		pH	0.1902	0.0010
		Humic substance	0.1264	0.0030
		Water content	0.0517	0.0370
		elevation	0.0650	0.0110
		Slope	0.0958	0.0140
	Old	Herb Shannon's diversity	0.0764	0.0010
		Associated tree Shannon's diversity	0.1199	0.0010
		ECM tree Shannon's diversity	0.2682	0.0010
		Available B	0.1614	0.0010
		Available Fe	0.1529	0.0010
		Exchangeable Cu	0.0763	0.0080
		Available P	0.0999	0.0010
		Available N	0.0942	0.0040
		Total N	0.1836	0.0010
		Organic matter	0.1390	0.0010
		pH	0.1770	0.0010
		Water content	0.1802	0.0010

Table S6 Multiple regression of distance matrices (MRM) of fungal pathogen community composition against abiotic and biotic predictors for young, intermediate and old forest

Responders	Forest age	Distance matrix	Coefficient	*P*	Model parameters
Phytopathogenic fungi	Young	ECM tree Shannon's diversity	0.0171	0.0352	R^2=0.2510893, *F*=84.39292, *P*=0.0001000
		Elevation	0.0178	0.0001	
		Available Mn	0.0110	0.0015	
		Available Fe	0.0186	0.0266	
		Tree Shannon's diversity	0.0079	0.0095	
		Associated tree weight	0.0155	0.0011	
		EMF	0.0345	0.0001	
	Intermediate	Plant Shannon's diversity	0.0118	0.0004	R^2=0.2576806, *F*=87.19252, *P*=0.0001000
		Elevation	0.0108	0.0004	
		Organic matter	0.0115	0.0008	
		pH	0.0211	0.0001	
		Total P	0.0199	0.0001	
		Exchangeable Ca	0.0057	0.0066	
		Available P	0.0061	0.0176	
		Available N	0.0136	0.0001	
		Available B	0.0121	0.0001	
		Tree weight	0.0078	0.0035	
		EMF	0.0255	0.0001	
	Old	ECM tree Shannon's diversity	0.0076	0.0003	R^2=0.2341217, *F*=97.85689, *P*=0.0001000
		Plant Shannon's diversity	0.0066	0.0006	
		Slope	0.0069	0.0005	
		Humic substances	0.0093	0.0002	
		pH	0.0099	0.0001	
		Quick acting K	0.0083	0.0090	
		Total K	0.0069	0.0006	
		Total N	0.0162	0.0001	
		Total P	0.0075	0.0016	
		Exchangeable Ca	0.0061	0.0022	
		Available P	0.0044	0.0366	
		Available N	0.0162	0.0001	
		Available Zn	0.0079	0.0246	
		Available B	0.0071	0.0006	
		Available Cu	0.0044	0.0102	
		Grass Shannon's diversity	0.0089	0.0004	
		EMF	0.0300	0.0001	
